# Sculpting the developing brain: a review of neural mechanisms linking physical activity to executive function and emotion regulation in youth

**DOI:** 10.3389/fpsyg.2026.1865879

**Published:** 2026-09-29

**Authors:** Deng-tai Wen, Ying Lin, Jing-yao Sun, Shou-zhi Lv, Ji-ying Wang, Tian-shuo Yuan, De-yu Zhu

**Affiliations:** Department of Physical Education, Ludong University, Yantai, Shandong, China

**Keywords:** children and adolescents, emotional brain regions, executive function, neural mechanisms, neuroplasticity, physical exercise

## Abstract

Childhood and adolescence are a critical period for brain development, yet global physical inactivity threatens this neurodevelopmental trajectory. This review synthesizes the past decade of evidence on how physical exercise modulates executive function and emotional brain circuitry in youth. Behaviorally, moderate-intensity aerobic exercise may enhance inhibitory control, working memory, and cognitive flexibility. However, effect sizes are consistently small-to-moderate, and not all cognitive domains show uniform benefits, with working memory effects being smaller and more variable, and high-intensity exercise sometimes impairing performance. Neuroimaging findings reveal enhanced prefrontal-amygdala functional connectivity, strengthening top-down emotion regulation. Mechanistically, acute exercise appears to enhance cognition via transient neurochemical and hemodynamic changes, while chronic exercise may induce longer-lasting neuroplasticity through BDNF-related pathways, although these mechanisms remain largely inferred and peripheral BDNF is an imperfect surrogate for central activity. Cognitively engaging activities yield superior benefits, with the most consistently supported protocols involving approximately 45-min sessions, three times weekly, sustained for 12 weeks or longer. These parameters represent modal characteristics of successful interventions rather than precisely established optimal prescriptions, and substantial heterogeneity across studies precludes highly specific, universally applicable recommendations. Notably, neurodevelopmental populations like ADHD exhibit distinct or amplified responsiveness. Key limitations include methodological heterogeneity, fragmented causal inference, small sample sizes in neuroimaging studies, and limited evidence for clinical populations. Future work must advance precision exercise prescriptions based on individual neural profiles and translational research bridging laboratory findings with real-world educational contexts.

## 1 Introduction

### 1.1 Problem statement: the critical period of brain development in children and adolescents and the significance of exercise intervention

Childhood and adolescence constitute a critical window for the structural and functional maturation of the brain, with dynamic processes including synaptic pruning, myelination, and functional network reorganization continuing well into young adulthood (Andujar et al., 2026; Chun et al., 2025; Di Vincenzo et al., 2026; Gogtay et al., 2004; Lenroot and Giedd, 2006; Leucht et al., 2025; Raduner et al., 2025; Soleimani-Rad et al., 2025; Sowell et al., 1999; Tiemeier et al., 2010; Yang and Shen, 2026). During this window, executive function – encompassing inhibitory control, working memory, and cognitive flexibility – exhibits rapid advancement, with inhibitory control maturing earliest and higher-order functions developing later (Anderson, 2002; Best and Miller, 2010; Diamond, 2020; Miyake et al., 2000). Emotional regulation similarly undergoes significant transformation during this period, as the developmental asynchrony between limbic system reactivity and prefrontal regulatory capacity contributes to heightened emotional reactivity and risk-taking behavior in adolescence (Ahmed et al., 2015; Blakemore and Choudhury, 2006; Casey et al., 2008; Garon et al., 2008; Myruski and Dennis-Tiwary, 2021; Zelazo and Carlson, 2012). These neurodevelopmental processes lay the foundation for lifelong cognitive and emotional health.

Nevertheless, this crucial neurodevelopmental trajectory is threatened by unprecedented contemporary challenges. According to the World Health Organization, 81% of adolescents aged 11–17 years globally fail to meet recommended physical activity levels (de Lima et al., 2026; Pasquerella et al., 2025). A systematic review by Poitras et al., based on objectively measured data, confirmed that only a minority of children and adolescents achieve the recommended 60 min of moderate-to-vigorous physical activity daily, with activity levels declining steadily with advancing age (Poitras et al., 2016). This behavioral pattern not only engenders physical health sequelae such as obesity and elevated cardiovascular risk but may also compromise the healthy development of cognitive and emotional systems by depriving the maturing brain of essential sensorimotor stimulation (Janssen and LeBlanc, 2010; Palmer et al., 2025; Strong et al., 2005).

As a highly malleable environmental stimulus, physical exercise has garnered unprecedented attention within the field of cognitive neuroscience of exercise for its salutary effects on brain health (Nuttall et al., 2026; Stillman and Erickson, 2018). Since the widespread adoption of neuroimaging techniques around 2012, the field has transitioned from descriptive to mechanistic investigations (Erickson et al., 2019; Huynh et al., 2023; Siregar et al., 2023). The central scientific inquiry has shifted from the binary question of whether exercise benefits the brain to the mechanistic interrogation of how exercise remodels the brain and which specific exercise modalities confer the greatest benefit to whom (Erickson et al., 2019; Siregar et al., 2023). This paradigm shift has elevated physical exercise from a generalized health promotion strategy to a precisely modifiable neurobehavioral intervention (Gogtay et al., 2004; Northey et al., 2025; Schmitt et al., 2014).

However, despite substantial progress, the evidence base is marked by considerable inconsistencies that warrant critical evaluation. While meta-analyses generally support positive effects of exercise on cognitive function, individual studies frequently report null or contradictory findings – particularly for working memory, high-intensity exercise, and BDNF-mediated mechanisms (Alvarez-Bueno et al., 2017; de Greeff et al., 2018; de Lucca et al., 2023; de Menezes-Junior et al., 2022; de Oliveira Segundo et al., 2024; Dinoff et al., 2016; Donnelly et al., 2016; Fernández-Rodríguez et al., 2022; Li et al., 2025a,c; Tao et al., 2025; Tutakhail et al., 2026). The magnitude of exercise effects varies considerably across cognitive domains (strongest for inhibitory control, weakest for working memory and cognitive flexibility), exercise intensities (moderate-intensity effects are most consistent; high-intensity findings are mixed), and outcome modalities (behavioral effects are more consistent than neuroimaging or biomarker effects) (Alvarez-Bueno et al., 2017; de Greeff et al., 2018; Dinoff et al., 2016; Donnelly et al., 2016; Li et al., 2025a,c; Tao et al., 2025). These inconsistencies have led to ongoing debates regarding the specificity, generalizability, and mechanistic basis of exercise-induced cognitive benefits (Erickson et al., 2019; Guzmán-Muñoz et al., 2025; Li et al., 2025a,c; Singh et al., 2025; Stillman et al., 2020; Unterguggenberger, 2025). Critically evaluating these conflicting findings – rather than selectively highlighting positive results – is essential for advancing the field toward a more nuanced understanding of the exercise-brain-behavior relationship. The present review systematically synthesizes the past decade of evidence on how physical exercise modulates executive function and emotional brain circuitry in youth, with explicit attention to both consistent findings and areas of divergence.

### 1.2 Definition of core concepts

The core constructs examined in this review require precise operational delineation to ensure conceptual consistency throughout the subsequent discussion.

Physical Exercise is operationalized herein across two distinct analytical levels: acute physical activity and chronic regular exercise. Acute exercise refers to a single, sustained bout of physical activity, typically lasting 20–60 min, and is employed to investigate the immediate, transient neurocognitive effects of movement (Chang et al., 2012; Ludyga et al., 2016). Chronic exercise denotes regular, structured exercise interventions spanning several weeks to months, utilized to examine cumulative structural and functional adaptations within the brain (Alvarez-Bueno et al., 2017; de Greeff et al., 2018; Donnelly et al., 2016). Exercise modalities encompass a spectrum including aerobic exercise, resistance training, coordinative exercise, and cognitively engaging physical activities (Zhang et al., 2026). Exercise intensity is stratified into three tiers – low, moderate, and high – based on heart rate reserve (HRR) or maximal oxygen uptake (VO_2_max). Moderate intensity typically corresponds to 60–70% of HRR or a rating of perceived exertion (RPE) of 12–14 on the Borg scale. Exercise frequency and duration are incorporated into the analytical framework as independent dosage dimensions.

To further clarify the conceptual landscape, it is useful to distinguish among six related but distinct constructs that are occasionally used interchangeably in the literature: Physical activity is any bodily movement produced by skeletal muscles requiring energy expenditure, encompassing activities of daily living and leisure-time pursuits. Exercise is a planned, structured, repetitive subcategory of physical activity with the specific objective of improving fitness; we distinguish acute exercise (single bout) from chronic exercise (regular training over weeks to months). Cardiorespiratory fitness is a physiological capacity – the ability to supply oxygen during sustained activity – influenced by genetics, maturation, and physical activity, but not synonymous with exercise behavior. Sports participation refers to organized, rule-governed physical activities that combine physical movement with social interaction, strategic decision-making, and emotional engagement. Movement is the broadest term, encompassing any change in body position, including incidental actions; in embodied cognition, movement is constitutive of cognitive and emotional experience. Cognitively engaging exercise embeds cognitive challenges – strategic decision-making, rule-switching, cooperative coordination – within the movement task, and is hypothesized to yield superior benefits through synergistic motor-cognitive network activation. Table 1 summarizes these six constructs.

**TABLE 1:** Conceptual distinctions among physical activity, exercise, cardiorespiratory fitness, sports participation, movement, and cognitively engaging exercise.

Construct	Definition	Primary characteristics	Measurement	Relationship to brain/cognition	Examples
Physical activity	Any bodily movement produced by skeletal muscles requiring energy expenditure	Broad behavioral category; includes all movement contexts	Accelerometry, self-report, pedometry	Broadly associated with brain health; context-dependent effects	Walking to school, climbing stairs, gardening, active commuting
Exercise	Planned, structured, repetitive physical activity with a specific objective	Subcategory of PA; purposive; acute vs. chronic distinction	Structured protocols, heart rate, duration/frequency	Most direct evidence for cognitive benefits; acute vs. chronic differ mechanistically	Aerobic running, stationary cycling, resistance training
Cardiorespiratory fitness	Physiological capacity to supply oxygen to muscles during sustained activity	Not a behavior; influenced by genetics, maturation, and PA	VO_2_max, heart rate recovery, fitness tests	Cross-sectional fitness-cognition associations; not equivalent to activity effects	High vs. low fitness groups (cross-sectional comparisons)
Sports participation	Engagement in organized, rule-governed physical activities	Combines PA with social interaction, rules, competition/cooperation	Self-report, team membership, hours per week	Unique socioemotional and cognitive flexibility benefits	Basketball, soccer, tennis, martial arts, dance
Movement	Any change in body position or orientation; broadest term	Encompasses all forms (volitional/non-volitional; gross/fine)	Direct observation, motion capture, accelerometry	Embodied cognition framework; movement as constitutive of experience	Postural adjustments, gesturing, incidental movement, fine motor actions
Cognitively engaging exercise	Physical activity that embeds cognitive challenges within the movement task	Active recruitment of executive processes during exercise	Task complexity ratings, cognitive load during activity	Superior EF benefits; synergistic motor-cognitive network activation	Dance with complex sequences, team sports with strategic demands, interactive exergames

PA, physical activity; EF, executive function; VO_2_max, maximal oxygen consumption. These constructs are conceptually distinct but often overlap in practice (e.g., a basketball game qualifies as physical activity, exercise, sports participation, and cognitively engaging exercise). The distinctions are important for interpreting evidence: findings from fitness-based cross-sectional studies do not necessarily generalize to exercise interventions, and benefits of sports participation may reflect social as well as physical mechanisms. Throughout this review, we use each term according to the definitions above.

Executive Function refers to a suite of higher-order cognitive processes that subserve goal-directed behavior. In accordance with the classic tripartite model, it is delineated into three core components: inhibitory control, working memory, and cognitive flexibility (Richter et al., 2023; Segura et al., 2024). Inhibitory control denotes the capacity to suppress prepotent responses or interfere with distracting information, encompassing two subcomponents: interference control and response inhibition. Working memory refers to the ability to transiently maintain and manipulate information, involving both online maintenance and active processing of mental representations. Cognitive flexibility denotes the capacity to adaptively shift between different tasks or mental sets and represents the latest-maturing and most hierarchically advanced facet of executive function. While these three facets are functionally distinct, they are intrinsically interrelated and collectively underpin complex cognitive operations such as planning, reasoning, and problem-solving (Bandettini et al., 2025; Hendry et al., 2016; Willoughby and Hudson, 2023). It is noteworthy that contemporary research increasingly conceptualizes executive function as a hierarchical structure wherein foundational inhibitory control and working memory constitute developmental prerequisites for cognitive flexibility (Bao et al., 2024).

It is important to acknowledge, however, that the tripartite model of executive function – while widely cited and heuristically valuable – is not without substantial debate, particularly when applied to pediatric populations. The influential framework proposed by Miyake et al. (2000), based on latent variable analysis in adults, identified three separable but correlated components (inhibitory control, working memory, and cognitive flexibility), establishing the ‘unity and diversity’ model of executive function (Miyake et al., 2000). However, the applicability of this framework to children and adolescents remains an active area of investigation. Developmental studies have yielded mixed findings regarding whether the three components are truly separable in younger populations, with some studies supporting a unitary structure in early childhood that progressively differentiates with age, and others suggesting that the components are distinguishable even in preschool-aged children (Best and Miller, 2010; Fernández-Rodríguez et al., 2022; Tutakhail et al., 2026).

Several additional complexities warrant acknowledgment. First, developmental measurement invariance – the assumption that executive function tasks measure the same construct across different age groups – has not been established for most commonly used EF tasks. A task that measures inhibitory control in a 10-year-old may tap different cognitive processes (e.g., processing speed, task comprehension, motor response efficiency) in a 6-year-old, complicating cross-age comparisons (Best and Miller, 2010; Dinoff et al., 2016). Second, task impurity poses a significant interpretational challenge: classic EF tasks such as the Stroop, Flanker, and N-back paradigms recruit multiple cognitive processes beyond their target EF component, including attentional orienting, response selection, and processing speed (Tutakhail et al., 2026). Observed exercise effects on a particular task cannot be unequivocally attributed to a single EF component. Third, the extent to which inhibitory control, working memory, and cognitive flexibility are supported by truly separable neural systems in younger populations remains an open question. While adult neuroimaging studies have identified distinct but overlapping networks for each component (e.g., prefrontal-anterior cingulate for inhibitory control, prefrontal-parietal for working memory, frontopolar-striatal for cognitive flexibility), developmental neuroimaging research suggests that these neural systems may be less differentiated in childhood, with greater functional overlap and less specialized recruitment (Segura et al., 2024; Tao et al., 2025; Tutakhail et al., 2026).

Throughout this review, we adopt the tripartite framework as a useful organizational heuristic, but we interpret findings regarding component-specific effects with appropriate caution, recognizing the conceptual and methodological complexities outlined above. Wherever possible, we note whether reported effects are specific to a given EF component or reflect more general cognitive enhancement.

Emotional Brain Regions, as defined within the scope of this review, primarily denote the core neural circuitry involved in emotion generation and emotion regulation. This network encompasses the amygdala (responsible for emotional salience detection and fear conditioning), the anterior cingulate cortex (ACC; implicated in emotional conflict monitoring and emotion regulation), the insula (subserving interoceptive processing and the somatic representation of emotional experience), the ventromedial prefrontal cortex (vmPFC; involved in the encoding of emotional value and decision-making), and the orbitofrontal cortex (OFC; participating in reward processing and the regulation of emotional expectancy) (Garon et al., 2008; Zelazo and Carlson, 2012). Of particular conceptual importance, the prefrontal cortex (PFC) is accorded a dual role in the present analysis. The dorsolateral PFC (dlPFC) primarily serves cognitive control functions within the executive domain, whereas the vmPFC and OFC are critically engaged in the top-down regulation of limbic structures during emotion regulation. This dual anatomical and functional role – “one structure, two functions” – constitutes a pivotal neural nexus wherein exercise concurrently exerts its influence on both cognitive and affective domains (Biddle et al., 2019; Lubans et al., 2016).

Neural Mechanisms refer to the multi-level biological pathways through which exercise influences the brain. This analytical framework spans three hierarchically organized strata: (a) the molecular and cellular level, encompassing the regulation of neurotrophic factor expression, alterations in neurotransmitter system sensitivity, and the induction and maintenance of synaptic plasticity; (b) the regional and circuit level, including changes in gray matter volume and cortical thickness, alterations in white matter microstructural integrity, enhanced efficiency of task-evoked activation patterns, and the reorganization of resting-state functional connectivity(rs-FC); and (c) the large-scale brain network level, involving functional integration and segregation both within and between distributed neural networks (Chaddock et al., 2010; Chaddock-Heyman et al., 2013; Huang et al., 2014; Stillman et al., 2020; Voss et al., 1985; Voss et al., 2011). This multi-level framework is intended to establish a comprehensive portrait of the exercise-brain-behavior causal continuum, spanning from microscopic molecular events to macroscopic network dynamics.

### 1.3 Research aims and review framework

The present review aims to systematically synthesize research advances concerning the neural mechanisms through which physical exercise influences executive function and emotional brain regions in youth. The review is organized along a logical continuum spanning “behavioral manifestation → regional localization → neural mechanisms → moderating factors → translational application” (Erickson et al., 2019; Guzmán-Muñoz et al., 2025; Stillman et al., 2020). Specifically, the objectives of this review are fourfold: (1) to integrate empirical findings across multiple dimensions, including acute versus chronic exercise, aerobic versus cognitively engaging physical activities, and typically developing versus neurodevelopmental disorder populations; (2) to elucidate the neural pathways through which exercise modulates executive function and emotion regulation across three hierarchical levels – molecular, regional, and network-level mechanisms; (3) to identify key variables that moderate exercise effects, encompassing both exercise prescription parameters and individual difference factors; and (4) to delineate future research directions and educational translational pathways based on the identified limitations of the extant evidence base. The structure of the full article is organized as follows: Section “2 Theoretical foundations and research paradigms” elucidates the theoretical foundations and research paradigms underlying the influence of exercise on brain development; Section “3 Physical exercise and executive function: effects and neural mechanisms “focuses on the effects of physical exercise on the three constituent components of executive function and their associated neural mechanisms; Section “4 Physical exercise and emotional brain regions: effects and neural mechanisms” examines the impact of physical exercise on emotion-related brain regions and the underlying mechanisms; Section “5 Moderating variables and boundary conditions” analyzes moderating variables and boundary conditions; Section “6 Research limitations and future directions” discusses research limitations and future directions; and Section “7 Conclusions and implications” synthesizes core conclusions and practical implications.

### 1.4 Literature search strategy

#### 1.4.1 Review type

Given the heterogeneity of study designs, outcome measures, and age ranges across the included literature, findings were synthesized narratively rather than meta-analytically. Accordingly, while the search and selection process followed systematic principles, the synthesis approach is narrative, making this a systematic narrative review – a systematic search and PRISMA-style selection process followed by a narrative synthesis of findings.

This narrative review employed a systematic literature search strategy to ensure transparency and reproducibility. The search was conducted on April 25, 2026 across four electronic databases: PubMed, Web of Science, Scopus, and the China National Knowledge Infrastructure (CNKI). The search timeframe was restricted to publications from January 1, 2012, to April 25, 2026, with the inclusion of earlier seminal works (2000–2011) identified through citation tracing and expert recommendation. The full Boolean search string used for PubMed was as follows: (“physical activity”[Mesh] OR “exercise”[Mesh] OR “physical fitness”[Mesh] OR “motor activity”[Mesh] OR physical activity[tiab] OR exercise[tiab] OR aerobic exercise[tiab] OR cognitively engaging exercise[tiab]) AND (“executive function”[Mesh] OR “cognitive control”[tiab] OR “inhibitory control”[tiab] OR “working memory”[tiab] OR “cognitive flexibility”[tiab] OR “emotion regulation”[tiab] OR “prefrontal cortex”[Mesh] OR “amygdale”[Mesh] OR “neuroimaging”[Mesh] OR “brain-derived neurotrophic factor”[Mesh] OR BDNF[tiab]) AND (“child”[Mesh] OR “adolescent”[Mesh] OR “pediatrics”[Mesh] OR children[tiab] OR adolescents[tiab] OR youth[tiab]). This strategy was adapted for Web of Science, Scopus, and CNKI using equivalent subject headings and syntax appropriate to each platform. For CNKI, search terms were translated into Chinese and combined using the same Boolean logic.

#### 1.4.2 Inclusion and exclusion criteria

Studies were included if they met all of the following criteria: (a) peer-reviewed original research (randomized controlled trials, quasi-experimental designs, longitudinal studies, or cross-sectional observational studies) or systematic reviews/meta-analyses; (b) examined the relationship between physical activity/exercise and cognitive, emotional, or neurobiological outcomes; (c) focused on typically developing children and/or adolescents (aged 5–17 years); studies involving clinical populations (e.g., ADHD, autism spectrum disorder) were also included when they provided relevant mechanistic insights into the effects of exercise on executive function or emotional brain circuitry that could inform understanding of typical development; and (d) published in English or Chinese

Studies were excluded if they: (a) were case reports, editorials, conference abstracts, or opinion pieces without original data; (b) focused exclusively on clinical populations (e.g., ADHD, depression, traumatic brain injury) without providing relevant mechanistic insights applicable to typical development; (c) involved adult participants only (≥18 years) with no pediatric subsample disaggregation; or (d) were not available in English or Chinese.

#### 1.4.3 Screening and selection process

The initial database search yielded 2,325 records. After removing duplicates, 1,899 unique records remained. Two authors independently screened titles and abstracts against the inclusion criteria, resulting in 431 full-text articles assessed for eligibility. Following full-text review, 271 articles were excluded for reasons including lack of pediatric subsample data and absence of neuroimaging or biomarker measures. Ultimately, 160 studies met the inclusion criteria and were included in the final synthesis. A PRISMA-style flow diagram illustrating this selection process is provided in Figure 1. Disagreements between reviewers at any stage were resolved through discussion or by consultation with a third author.

**FIGURE 1:**
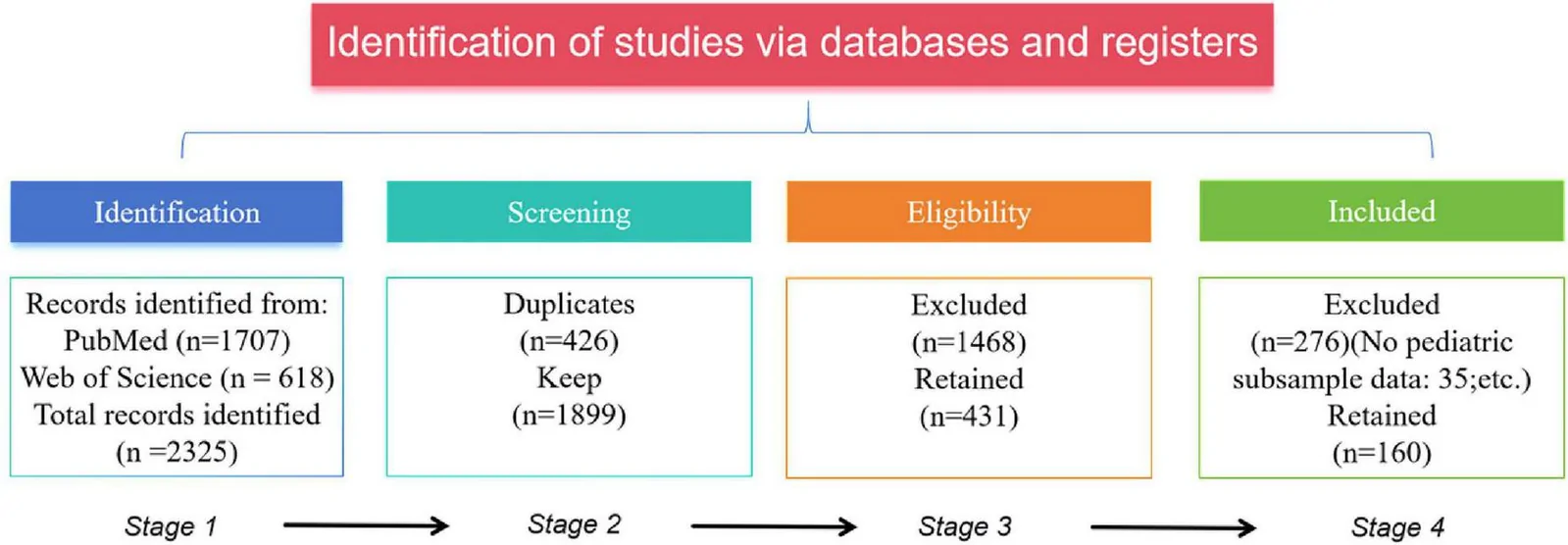
PRISMA flow diagram of the literature search and screening process. A systematic search was conducted across four databases (PubMed, Web of Science, Scopus, and CNKI), yielding a total of 2,325 records. After removing duplicates, 1,899 records remained. Title and abstract screening excluded 1,468 records as not relevant to the topic, leaving 431 records for full-text assessment. Following full-text review, 271 articles were excluded for reasons including lack of pediatric subsample data and absence of neuroimaging or biomarker measures. Ultimately, 160 studies met the inclusion criteria and were included in the final synthesis.

#### 1.4.4 Data extraction and synthesis

From each included study, we extracted data on study design, sample characteristics, exercise intervention parameters (type, intensity, duration, frequency), outcome measures (cognitive, emotional, neuroimaging, biomarker), and main findings. Given the heterogeneity of study designs, outcome measures, and age ranges, findings were synthesized narratively rather than meta-analytically. Evidence was stratified by study design (animal models, human correlational neuroimaging, randomized interventions) to distinguish causal from associational inferences, as detailed in the Discussion section.

#### 1.4.5 Evidence grading approach

To facilitate interpretation of the evidence, we adopted a GRADE-inspired confidence rating framework for major conclusions and mechanistic pathways (Tables 2, 3, and 4). Confidence levels (High/Moderate/Low/Very Low) were assigned based on qualitative assessment of the following criteria: (a) the number and design of supporting studies, with greater weight given to meta-analyses and randomized controlled trials; (b) the consistency of findings across independent investigations; (c) the presence and magnitude of statistical heterogeneity (I^2^) where reported in meta-analyses; (d) the directness of the evidence to the pediatric population and outcome of interest; and (e) risk of bias, considered qualitatively based on sample size, study design, and methodological quality as reported in the original studies. These ratings were discussed and agreed upon by two authors. It is important to note that this grading is intended to provide a concise summary of evidence strength for readers and is not a substitute for a formal, comprehensive risk-of-bias assessment of each included study, which was beyond the scope of this narrative review (Figure 1).

**TABLE 2:** Evidence grading for exercise prescription parameters in youth.

Parameter	Recommendation / modal value	Supporting evidence	Consistency of findings	Key heterogeneity	Confidence level*
Modality	Cognitively engaging physical activity (CEPA) > aerobic alone	Multiple meta-analyses (Teng et al., 2025; Mao et al., 2024; Vazou et al., 2019)	Moderate to high; CEPA consistently shows superior effect sizes across studies	Variation in definition of “cognitive engagement”; studies differ in CEPA operationalization	⨁⨁⨁° Moderate
Intensity	Moderate (60–70% HRR)	Multiple meta-analyses (Chang et al., 2012; Ludyga et al., 2016; Li et al., 2025a)	Moderate; inverted U-shape consistently observed, but specific intensity ranges vary	HRR calculation methods differ; fitness level modifies optimal intensity	⨁⨁⨁° Moderate
Session Duration	∼45 min	Multiple studies; few direct comparisons	Low to moderate; most positive studies use 30–45 min, but no controlled dose-response comparisons	Age, fitness, modality, and outcome all moderate duration effects; exact 45-min threshold arbitrary	⨁⨁°° Low
Frequency	≥3 sessions/week	Singh et al. (2019) meta-analysis; expert consensus	Moderate; most positive protocols use ≥ 3 × /week, but frequency is often confounded with other parameters	Interaction with intensity and duration; no controlled frequency-comparison studies	⨁⨁⨁° Moderate
Intervention Length	≥12 weeks	Multiple meta-analyses (de Greeff et al., 2018; Alvarez-Bueno et al., 2017; Donnelly et al., 2016; Singh et al., 2019)	Moderate to high; longer interventions generally associated with larger effects	Outcomes differ: structural changes require longer; functional changes may emerge earlier	⨁⨁⨁° Moderate
Cognitive Outcome	Inhibitory control > working memory > cognitive flexibility	Consistent across meta-analyses	High; inhibitory control consistently shows largest effect sizes	Sensitivity may reflect task properties as well as neural mechanisms	⨁⨁⨁⨁ High

*Confidence levels adapted from GRADE framework: ⨁⨁⨁⨁ High = consistent evidence from multiple well-designed studies; ⨁⨁⨁° Moderate = consistent evidence but with heterogeneity or methodological limitations; ⨁⨁°° Low = limited evidence with notable heterogeneity or indirectness; ⨁°°° Very Low = insufficient or highly inconsistent evidence. The parameters listed represent the modal characteristics of successful interventions identified across existing studies rather than precisely established optimal prescriptions. The confidence level reflects the current state of evidence and should guide interpretation of each recommendation.

**TABLE 3:** Evidence quality assessment for major conclusions in the review.

Major conclusion	Supporting study types	Methodological quality / risk of bias	Publication bias	Heterogeneity (I^2^)	Sample size limitations	Certainty of evidence*
Acute exercise → improved inhibitory control	Meta-analyses (Chang et al., 2012; Ludyga et al., 2016); multiple RCTs	Moderate; most studies use within-subject designs with no blinding; high variability in exercise protocols	Likely present; meta-analyses show funnel plot asymmetry in some comparisons	Moderate to high (I^2^ often 50–70%)	Adequate for meta-analyses; individual studies often n < 30 per condition	⨁⨁⨁° Moderate
Chronic exercise ( ≥ 12 weeks) → improved inhibitory control	Meta-analyses (de Greeff et al., 2018; Alvarez-Bueno et al., 2017; Singh et al., 2019); large RCTs (FITKids, Davis et al., 2011)	Moderate to good; several high-quality RCTs with active control groups, but blinding of participants impossible	Likely present; funnel plots suggest some asymmetry	Moderate (I^2^ ∼40–60%)	RCTs often n = 50–100 per group; adequate for moderate effects	⨁⨁⨁° Moderate
Chronic exercise → improved working memory	Meta-analyses (de Greeff et al., 2018; Singh et al., 2019); RCTs	Moderate; fewer high-quality RCTs specifically targeting working memory; task heterogeneity	Insufficient data to assess	Moderate to high (I^2^ often > 60%)	Often underpowered for working memory sub-analyses	⨁⨁°° Low
Chronic exercise → improved cognitive flexibility	Meta-analyses (Vazou et al., 2019; Teng et al., 2025); multiple RCTs	Moderate; evidence concentrated in cognitively engaging exercise studies; task variability	Insufficient data to assess	Moderate to high (I^2^ ∼50–65%)	Limited by number of studies; task-switching paradigms vary widely	⨁⨁°° Low
Exercise → enhanced prefrontal-amygdala connectivity (neuroimaging)	Cross-sectional rs-fMRI studies; few RCTs with pre/post imaging (Krafft et al., 2014; FITKids subsample)	Low to moderate; predominantly correlational; most studies lack pre/post design with exercise intervention; no blinding	Likely present; negative findings underreported	Not applicable (insufficient studies for meta-analysis)	Neuroimaging studies typically n = 20–40; severely underpowered	⨁°°° Very Low
Exercise → BDNF elevation	Meta-analyses (Szuhany et al., 2015; Dinoff et al., 2016; de Menezes-Junior et al., 2022)	Moderate; meta-analyses include heterogeneous studies; BDNF assay methods vary; peripheral vs. central BDNF not distinguished	Likely present; funnel plots show some asymmetry for acute exercise studies	High for acute exercise (I^2^ often > 70%); lower for chronic interventions (I^2^ ∼40–50%)	Meta-analyses have adequate power, but individual studies often small	⨁⨁⨁° Moderate (peripheral BDNF) / ⨁°°° Very Low (central BDNF)
Cognitively engaging exercise > aerobic exercise alone	Network meta-analysis (Teng et al., 2025; Mao et al., 2024); head-to-head comparisons	Moderate; few direct comparisons; most evidence from indirect comparisons; “cognitively engaging” definitions vary	Insufficient data to assess	Moderate (I^2^ ∼40–55%)	Limited number of head-to-head comparison trials	⨁⨁°° Low
ADHD population shows amplified exercise effects	Meta-analyses (Cerrillo-Urbina et al., 2015; Den Heijer et al., 2017; Liu Y. et al., 2025)	Moderate; small number of RCTs; heterogeneity in outcomes and exercise protocols; ADHD diagnosis variability	Likely present; small-study effects possible	Moderate to high (I^2^ often > 60%)	Limited by small number of studies	⨁⨁°° Low
Exercise → reduced depression/anxiety in youth	Umbrella review (Biddle et al., 2019); meta-analyses (Carter et al., 2016; Bailey et al., 2018; Singh et al., 2026)	Moderate; multiple RCTs but heterogeneity in depression measures and exercise protocols; low risk of detection bias	Likely present; funnel plot asymmetry in some analyses	Moderate to high (I^2^ often 50–70%)	Large meta-analyses have adequate power	⨁⨁⨁° Moderate
Embodied cognition framework integration	Theoretical synthesis; indirect support from cognitively engaging exercise studies	Not applicable (theoretical framework)	Not applicable	Not applicable	Not applicable	⨁°°° Very Low (theoretical synthesis)

Adapted from GRADE framework. Certainty ratings reflect the confidence that the observed effect is real and that the conclusion is likely to hold across populations and contexts. *Confidence levels: ⨁⨁⨁⨁ High = consistent evidence from multiple well-designed studies with low heterogeneity; ⨁⨁⨁° Moderate = consistent evidence but with heterogeneity or methodological limitations; ⨁⨁°° Low = limited evidence with notable heterogeneity or risk of bias; ⨁°°° Very Low = insufficient or highly inconsistent evidence; theory-based conclusions have inherently lower empirical confidence levels.

**TABLE 4:** Evidence grading for mechanistic pathways in the exercise-brain-behavior cascade.

Mechanistic link	Source of evidence	Directly demonstrated in humans?	Causal evidence established?	Confidence level*
Exercise → Peripheral BDNF ↑	Human meta-analyses (Szuhany et al., 2015; Dinoff et al., 2016); RCTs	Yes (peripheral blood measures)	Yes (exercise intervention → peripheral BDNF elevation)	⨁⨁⨁⨁ High
Exercise → Central (Brain) BDNF ↑	Animal models (Vaynman et al., 2004); inferred from peripheral-correlation studies	No (no direct human brain BDNF measures in pediatric exercise studies)	No (peripheral-central correspondence not established)	⨁°°° Very Low
BDNF → TrkB → MAPK/ERK/PI3K-Akt signaling	Animal models (Vaynman et al., 2004; Knaepen et al., 2010)	No (invasive assays not feasible in humans)	Yes (in animal models); No (in humans)	⨁⨁°° Low (human inference)
BDNF → Synaptic plasticity (LTP, spine density)	Animal models (*in vitro* and *in vivo* rodent studies)	No (not feasible in human children)	Yes (in animals); No (in humans)	⨁⨁°° Low (human inference)
Synaptic plasticity → Frontoparietal network efficiency	Theoretical synthesis; inferred from correlational neuroimaging (rs-FC, fMRI)	No (correlational only; no direct test of synaptic-to-network link)	No (purely inferential)	⨁°°° Very Low
Frontoparietal network efficiency → Executive function ↑	Correlational neuroimaging (rs-FC, task-fMRI; Voss et al., 2011; Chaddock-Heyman et al., 2013; Cline et al., 2024)	Yes (correlational, not causal); no RCTs directly test mediation	No (rs-FC changes are correlates, not established mechanisms)	⨁⨁°° Low
Exercise → Cerebral blood flow/prefrontal oxygenation ↑	Human fNIRS studies (Mücke et al., 2018; Du et al., 2025); acute exercise RCTs	Yes (directly measured in humans)	Yes (acute exercise → increased oxygenation); No (chronic adaptations → cognition)	⨁⨁⨁° Moderate (acute) / ⨁⨁°° Low (chronic-cognition link)
Exercise → VEGF ↑→ Angiogenesis → Gray/white matter integrity	Animal models (VEGF upregulation); human cross-sectional neuroimaging (DTI; Chaddock-Heyman et al., 2014a; Schaeffer et al., 2014)	Partial (structural associations in humans are correlational)	No (cross-sectional associations; causation not established)	⨁⨁°° Low
Exercise → Prefrontal-amygdala connectivity ↑→ Emotion regulation ↑	Cross-sectional fMRI (human correlational); animal models (BDNF-dependent myelination)	Yes (correlational, not causal); no RCTs directly test mediation	No (correlational; alternative explanations not ruled out)	⨁⨁°° Low
Exercise → Interoceptive accuracy ↑→ Insula activation ↑→ Emotion regulation ↑	Human correlational studies (interoception); theoretical framework (embodied cognition)	Yes (correlational interoceptive accuracy improvements); No (causal mediation not tested)	No (suggestive but unconfirmed; embodied cognition predictions untested)	⨁°°° Very Low
Cognitively engaging exercise → Superior EF gains	Meta-analyses (Teng et al., 2025; Mao et al., 2024); RCT head-to-head comparisons	Yes (behavioral RCTs)	Yes (behavioral level); No (mechanism level—whether cognitive engagement activates distinct neural pathways is inferred)	⨁⨁⨁° Moderate (behavioral) / ⨁⨁°° Low (mechanistic)
Exercise → Dopamine/NE release → Cognitive enhancement	Animal models; human pharmacological studies (indirect); PET studies (limited in pediatrics)	Partial (indirect pharmacological evidence); No (direct PET studies in children are rare)	No (causal role of dopamine/NE in exercise-cognition link not established in humans)	⨁°°° Very Low

This table provides a systematic evaluation of the evidence supporting each mechanistic link in the proposed exercise-brain-behavior cascade. Confidence levels are adapted from the GRADE framework. High (⨁⨁⨁⨁) = consistent evidence from multiple well-controlled human studies (RCTs or meta-analyses) directly testing the link. Moderate (⨁⨁⨁°) = consistent evidence but with methodological limitations or indirectness. Low (⨁⨁°°) = limited evidence with notable indirectness or reliance on animal models. Very Low (⨁°°°) = insufficient evidence; primarily theoretical or speculative. Key distinction: A link may be “established” at the behavioral level (e.g., exercise intervention → cognitive improvement) but not established at the mechanistic level (e.g., BDNF → executive function). Readers are encouraged to consult this table when interpreting the causal status of any pathway discussed in the text.

## 2 Theoretical foundations and research paradigms

### 2.1 Theoretical evolution in the cognitive neuroscience of exercise: from mind-body dualism to embodied cognition

The development of the cognitive neuroscience of exercise has undergone a profound theoretical shift from “mind-body dualism” toward “embodied” cognition (Stillman et al., 2020; Voss et al., 1985). Traditional cognitive psychology tended to conceptualize cognition as an abstract, symbolic processing system detached from the physical body, wherein exercise was relegated to an ancillary role – merely facilitating increased cerebral blood flow and elevated arousal levels. The seminal review published by Hillman et al. in Nature Reviews Neuroscience provided the first systematic articulation of an integrated ‘exercise-cognition-brain’ research framework, elevating the relationship among cardiorespiratory fitness, brain health, and cognitive function to a theoretical plane (Pontifex et al., 2011). Nevertheless, theoretical advances over the past decade have increasingly emphasized the active, constructive role of the body in shaping cognitive and emotional experience (Li et al., 2025a,c).

It is important to clarify the role that embodied cognition theory plays in this review. We deploy embodied cognition as a theoretical framework for interpreting and organizing existing findings, rather than as a directly tested empirical hypothesis. The framework provides a conceptual lens for understanding why certain types of physical activity – particularly those involving cognitive challenge, strategic decision-making, and coordinative demands – may confer unique cognitive and emotional benefits beyond those of purely repetitive aerobic exercise. As a theoretical framework, embodied cognition generates specific, empirically testable predictions: for example, interventions that enhance interoceptive awareness and sensorimotor integration should produce larger effects on emotion regulation than those that do not; and the cognitive benefits of exercise should be more pronounced when the movement task directly recruits executive processes. However, these predictions have not been directly tested in pediatric populations, and the evidence reviewed below should be understood as consistent with, but not confirmatory of, embodied cognition predictions. Embodied cognition theory posits that cognitive processes are fundamentally grounded in the sensorimotor experience of the body. The structural characteristics of the body, its action capabilities, and its movement history are not mere “containers” or “carriers” of cognition but rather constitutive elements of the cognitive process itself (Vorraber Lawson et al., 2025; Xu et al., 2025).

The concept of “deliberate play,” articulated by Pesce et al., provides an intervention design framework that is consistent with embodied cognition principles. Pesce et al. proposed that exercise interventions incorporating cognitive engagement, active decision-making, and environmental adaptation may yield superior executive function benefits compared with repetitive, automatized exercise (Pesce et al., 2016). It is important to note, however, that deliberate play was not designed as a direct test of embodied cognition theory; rather, it operationalizes a set of design principles that align with embodied predictions. Similarly, a meta-analysis by Vazou et al. found that, compared with purely repetitive exercise routines, physical activities incorporating cognitive challenges yield significantly more pronounced benefits for cognitive function, with effect sizes exceeding those of traditional protocols by an average of 0.2 to 0.3 standard deviations (Vazou et al., 2019). These findings are consistent with embodied cognition predictions – specifically, that tasks recruiting sensorimotor and cognitive processes jointly should produce larger cognitive gains – but they do not directly test the core tenets of embodied cognition (e.g., whether bodily states constitute cognitive content or whether sensorimotor experience is necessary for cognitive development). Tomporowski et al. (2015) systematically delineated the relationship between exercise characteristics and cognitive benefits, advancing the cognitive stimulation hypothesis of exercise. The cognitive stimulation hypothesis shares conceptual overlap with embodied cognition – both emphasize the active role of movement in shaping cognition – but it is a distinct, more circumscribed hypothesis that has been more directly tested in exercise intervention research. Investigators are no longer content to merely report the phenomenon that “cognitive performance improves after exercise”; instead, they now pursue more nuanced inquiries: Which types of physical activity experience – rule-governed ball sports versus rhythmically regulated aerobic running, or strategically demanding games characterized by cognitive challenge versus automatized repetitive movements – exert differential effects on specific cognitive components? How does the sense of physical competence and mastery acquired through movement mediate the effects of exercise on self-concept and emotional well-being? (Diamond and Ling, 2016; González-Del-Castillo and Barbero-Alcocer, 2025). Such inquiries have propelled the cognitive neuroscience of exercise beyond descriptive research toward mechanistic investigation, and from the broad proposition that “exercise is beneficial” toward the precision construction of “exercise prescriptions” (Singh et al., 2025; Unterguggenberger, 2025).

Diamond further delineated the critical ingredients for successful exercise interventions, contending that the effects of purely repetitive aerobic exercise are limited, whereas exercise programs that integrate cognitive challenge, social interaction, and emotional engagement (e.g., team sports, martial arts, dance) yield the most substantial benefits for executive function (Diamond, 2015). This perspective resonates with the “sensitive period hypothesis” advanced by Naylor et al., which holds that the rapid development of the PFC during childhood and adolescence renders this population particularly responsive to cognitively engaging forms of exercise (Northey et al., 2025). This developmental window may thus afford a unique opportunity for exercise interventions to produce maximally enduring and beneficial effects.

### 2.2 Principal research paradigms and technical approaches

#### 2.2.1 Key terminology note for interdisciplinary readers

To facilitate accessibility for readers from diverse disciplinary backgrounds, we provide brief definitions of key neuroscience terms that recur throughout this review:

BDNF (brain-derived neurotrophic factor) – a protein that supports the survival, growth, and synaptic plasticity of neurons, particularly in the hippocampus and PFC. It is a key mediator of exercise-induced neuroplasticity and is often measured in peripheral blood as a proxy for central neurotrophic activity (though with important limitations; see Section “2.3.1 Interpretational note on peripheral BDNF as a surrogate marker”).

DMN (default mode network) – a set of brain regions (including the medial PFC, posterior cingulate cortex, and angular gyrus) that show greater activity during rest, mind-wandering, and self-referential thought than during externally focused cognitive tasks. Exercise-related changes in DMN connectivity are interpreted as potential correlates of improved cognitive control.

TrkB (tropomyosin receptor kinase B) – the primary cell-surface receptor through which BDNF exerts its effects on neurons. When BDNF binds to TrkB, it triggers intracellular signaling cascades that promote neuronal survival, synaptic plasticity, and memory formation.

MAPK/ERK (mitogen-activated protein kinase/extracellular signal-regulated kinase) – a signaling pathway downstream of TrkB that transmits signals from the cell surface to the nucleus, regulating gene expression involved in synaptic plasticity and neuronal survival. It is one of the key molecular cascades through which BDNF promotes neuroplasticity.

PI3K/Akt (phosphoinositide 3-kinase/protein kinase B) – another signaling pathway downstream of TrkB that promotes cell survival and protein synthesis, complementing the MAPK/ERK pathway in BDNF-mediated neuroplasticity.

Graph-theoretical network measures – mathematical metrics used to characterize the organizational properties of brain networks derived from neuroimaging data. Common measures include: global efficiency (the average shortest path length between any two nodes, reflecting overall network integration), local efficiency (network clustering, reflecting segregation or specialization), and modularity (the degree to which a network can be divided into distinct, functionally specialized subnetworks). These measures provide quantitative descriptions of how efficiently brain regions communicate.

Functional segregation – the degree to which brain networks are organized into distinct, specialized subnetworks (modules) that process specific types of information. High functional segregation is generally interpreted as reflecting efficient, specialized neural processing.

Anticorrelation – a pattern of functional connectivity in which activity in one brain region or network consistently decreases while activity in another increases (negative correlation). A classic example is the anticorrelation between the DMN (active during rest) and the frontoparietal control network (active during focused cognitive tasks), which is thought to reflect efficient switching between internally and externally oriented processing.

Neural efficiency – a theoretical construct referring to the optimal use of neural resources: efficient neural processing is characterized by precise, focal activation of task-relevant regions combined with complete deactivation of task-irrelevant regions (often operationalized as more focal functional magnetic resonance imaging (fMRI) activation patterns or reduced neural effort for the same behavioral performance). It is important to note that “neural efficiency” is an inference from neuroimaging data, not a directly measured property; alternative interpretations (e.g., compensatory recruitment, reduced inhibitory tone) remain plausible.

Research in the cognitive neuroscience of exercise integrates a diverse array of methodological approaches – including behavioral measurement, brain imaging, neuromodulation, and molecular biology – thereby establishing a multi-level, multimodal methodological landscape (Li et al., 2025a).

Behavioral Measurement Paradigms primarily rely on classical cognitive tasks to assess the constituent components of executive function. The Flanker task and Stroop task are employed to measure inhibitory control, wherein interference control capacity is quantified by comparing reaction time differences between congruent and incongruent trials. The N-back task assesses working memory by manipulating memory load (e.g., 1-back, 2-back, 3-back) to examine the efficiency of online information maintenance and updating. Cognitive flexibility is evaluated using task-switching paradigms, wherein set-shifting ability is indexed by comparing reaction time differences between switch trials and repeat trials – termed the “switch cost” (Bull et al., 2020; Pratt, 2021). Emotion assessment adopts a combined approach utilizing standardized rating scales (e.g., Children’s Depression Inventory, State-Trait Anxiety Inventory, Positive and Negative Affect Schedule) and experimental paradigms (e.g., emotional face-matching task, emotional Stroop task) (Erickson et al., 2019; Northey et al., 2025). Academic achievement, as a distal outcome measure, is typically incorporated as a supplementary indicator using standardized test scores or teacher ratings.

Brain Imaging Techniques constitute the core toolkit for elucidating exercise-brain associations. Event-related potentials (ERPs), leveraging their millisecond-level temporal resolution, capture the fine-grained temporal dynamics of cognitive processing. P300 amplitude reflects the efficiency of attentional resource allocation and stimulus evaluation speed, whereas its peak latency indexes the time course of cognitive processing. The N200 component reflects conflict monitoring and the early stages of response inhibition, while the error-related negativity (ERN) reflects sensitivity to error detection and performance monitoring. Together, these components delineate the temporal dynamics of cognitive control (Byman et al., 2026; Drollette et al., 2025; Ludyga et al., 2024). Functional near-infrared spectroscopy (fNIRS), owing to its portability and compatibility with movement paradigms, is widely employed for monitoring prefrontal oxygenation, enabling real-time tracking of prefrontal cortical hemodynamic changes both during and following exercise (Chen et al., 2024; Mücke et al., 2018). Structural magnetic resonance imaging (sMRI) permits quantification of gray matter volume, cortical thickness, and white matter microstructural integrity. Voxel-based morphometry (VBM) is utilized to detect volumetric changes in gray matter, while diffusion tensor imaging(DTI) is employed to assess fractional anisotropy and radial/axial diffusivity within white matter fiber tracts (Lin et al., 2025; Liu Y. et al., 2025; Wang et al., 2025). Task-based fMRI (task-fMRI) reveals patterns of regional brain activation and effective connectivity during specific cognitive operations. Resting-state fMRI (rs-fMRI) characterizes intrinsic functional connectivity (rs-FC) through correlations in spontaneous BOLD signal fluctuations. rs-FC is a correlational metric reflecting statistical dependencies in neural activity; increased connectivity does not directly index ‘neural efficiency’ or ‘cognitive control’ – it may reflect myelination, synaptic strengthening, compensatory recruitment, or physiological artifacts, and is highly dependent on preprocessing decisions. Throughout this review, we interpret rs-FC findings as correlates requiring behavioral validation. (Chaddock-Heyman et al., 2014a; van Oort et al., 2023; Voss et al., 2011). Neuromodulation techniques, such as transcranial magnetic stimulation (TMS) and transcranial electrical stimulation (tES), are increasingly being deployed to verify causal pathways underlying exercise effects – specifically, by transiently enhancing or inhibiting activity within circumscribed brain regions to examine their interaction with exercise-induced outcomes.

Molecular Biological Indicators constitute an additional level of analysis. Peripheral brain-derived neurotrophic factor (BDNF) represents the most frequently assayed neurotrophin, with the Val66Met single-nucleotide polymorphism having been demonstrated to modulate exercise-induced BDNF release and associated cognitive benefits. Insulin-like growth factor-1 (IGF-1) is implicated in exercise-induced angiogenesis and neurogenesis, while vascular endothelial growth factor (VEGF) mediates cerebrovascular adaptations following exercise. Catecholaminergic neurotransmitters (dopamine, norepinephrine) and cortisol, as biomarkers of stress and arousal, are also incorporated into select study designs (de Azevedo et al., 2019; de Menezes-Junior et al., 2022; Kaufman et al., 2023; Rico-González et al., 2025a; Rico-González et al., 2025b; Tutakhail et al., 2026).

### 2.3 Macro-level pathway model of exercise effects on brain function

The pathways described below integrate findings from multiple lines of evidence, including animal models, human cross-sectional neuroimaging, randomized exercise interventions, and theoretical synthesis. It is important to note that these sources of evidence differ substantially in their capacity to support causal inferences. Evidence from randomized controlled trials supports causal claims at the behavioral level (i.e., exercise intervention → improved cognitive performance). Evidence from animal models provides direct mechanistic evidence at the molecular level but requires cautious extrapolation to humans. Evidence from human neuroimaging studies is predominantly correlational and cannot establish causation between exercise and neural changes. The integrated pathway model presented below should therefore be understood as a working hypothesis that synthesizes converging lines of evidence, rather than a fully established biological cascade (Evidence tier: Theoretical synthesis integrating animal models, human neuroimaging, and RCTs; working hypothesis; see Table 2). Wherever possible, we distinguish the strength of evidence supporting each proposed link.

Synthesizing the extant evidence, the macro-level pathways through which exercise influences brain function in youth can be conceptualized as a dual-track model comprising an acute effect pathway and a chronic effect pathway. These two pathways, while distinct in temporal scale, underlying mechanisms, and functional manifestations, are reciprocally interconnected and sequentially integrated (Guzmán-Muñoz et al., 2025; Li et al., 2025a).

The Acute Effect Pathway delineates the immediate impact of a single bout of exercise on brain function. Its core mechanisms operate at two interrelated levels: neurochemical and hemodynamic. At the neurochemical level, animal models and human correlational studies suggest that exercise may activate the locus coeruleus-norepinephrine system, potentially enhancing cortical arousal and the signal-to-noise ratio of neural processing, while also facilitating dopamine release that may augment reward and motivational system function (Cabral et al., 2026; Guzmán-Muñoz et al., 2025). At the hemodynamic level, exercise increases cardiac output and cerebral blood flow. fNIRS monitoring has demonstrated significant elevations in prefrontal oxyhemoglobin concentration immediately following exercise cessation (Hillman et al., 2009; Pontifex et al., 2011). A meta-analysis by Chang et al., encompassing 79 studies, confirmed that the overall effect size of acute exercise on cognitive performance ranges from small-to-moderate (g = 0.2–0.5; small-to-moderate by conventional benchmarks, with 95% confidence intervals not reported in the original meta-analysis), with peak effects manifesting approximately 11–20 min post-exercise. However, substantial heterogeneity was present across studies (I^2^ values ranging from 50% to 70%), indicating that the magnitude of acute exercise effects varies considerably as a function of exercise intensity, cognitive task, and participant characteristics (Chang et al., 2012). A further meta-analysis by Ludyga et al. revealed that the facilitative effects of moderate-intensity exercise on executive function are the most stable, whereas high-intensity exercise may transiently impair performance due to accumulated fatigue. Event-related potential (ERP) studies have characterized this effect along the temporal dimension (Ludyga et al., 2016). Drollette et al. reported that acute exercise elicits increased P300 amplitude and shortened P300 latency during inhibitory control tasks, indicative of enhanced attentional resource allocation efficiency and accelerated cognitive processing speed (Drollette et al., 2014; Drollette et al., 2025). This pathway exerts its influence from the immediate post-exercise period up to several hours thereafter, with effects gradually returning to baseline after approximately 60 min.

The Chronic Effect Pathway describes the enduring remodeling of brain structure and function engendered by long-term, regular exercise. Its core mechanisms involve the cumulative upregulation of neurotrophic factors and the sustained optimization of neural network efficiency. At the molecular level, repeated exercise stimuli are associated with cumulative increases in peripheral BDNF levels, as demonstrated by meta-analytic evidence. In animal models, exercise-induced muscle contraction triggers the release of fibronectin type III domain-containing protein 5 (FNDC5)/irisin, which has been shown to cross the blood-brain barrier and activate BDNF transcription in the rodent brain. This pathway remains largely inferred in humans, as direct evidence for FNDC5/irisin crossing the human BBB or for peripheral BDNF elevation reflecting central BDNF changes is currently lacking (de Lucca et al., 2023; de Menezes-Junior et al., 2022; de Oliveira Segundo et al., 2024; Fernández-Rodríguez et al., 2022; Tutakhail et al., 2026). A review by Huang et al. confirmed that regular exercise significantly elevates peripheral BDNF levels in healthy populations – an observation that, while important, should not be conflated with central neuroplasticity. Recognizing that peripheral BDNF is an imperfect surrogate for brain BDNF, we interpret these findings as correlates of exercise engagement rather than as direct evidence of exercise-induced brain neurotrophic changes. (Huang et al., 2014). A meta-analysis by Szuhany et al., aggregating 29 studies, verified a significant effect of exercise training on BDNF elevation (Hedges’ g = 0.46, 95% CI: 0.31–0.61; a small-to-moderate effect by conventional benchmarks). Heterogeneity in this meta-analysis was moderate (I^2^ ≈ 45%), suggesting reasonably consistent effects across studies (Szuhany et al., 2015). Dinoff et al. (2016) further demonstrated that high-intensity interval training (HIIT) elicits superior BDNF upregulation compared with moderate-intensity continuous exercise (g = 0.52, 95% CI: 0.38–0.66; a moderate effect), although the number of studies comparing HIIT to moderate-intensity exercise directly is limited and heterogeneity was notable. Jeon and Ha (2017) validated the dose-response relationship between exercise intensity and BDNF release in adolescent populations. A recent systematic review focusing on pediatric cohorts confirmed that neuromotor training or martial arts programs conducted at least three times per week for a minimum of 12 weeks significantly elevate BDNF levels (Rico-González et al., 2025a). Animal models have provided crucial mechanistic evidence: Vaynman et al. demonstrated that exercise-induced BDNF upregulation may activate the MAPK/ERK and PI3K/Akt signaling pathways via tropomyosin receptor kinase B (TrkB, the primary receptor through which BDNF signals) receptor activation, thereby promoting hippocampal synaptic plasticity, neurogenesis, and cell survival (Vaynman et al., 2004).

It is important to note, however, that the BDNF literature is not uniformly positive. Several studies have reported null or contradictory findings regarding exercise-induced BDNF elevation. For example, some individual RCTs have failed to find significant BDNF increases following exercise interventions, particularly when blood samples were collected at suboptimal time points (e.g., >2 h post-exercise) or when using plasma rather than serum assays (de Azevedo et al., 2019; Kaufman et al., 2023; Mücke et al., 2018). A systematic review by de Menezes-Junior et al. (2022) noted that while meta-analytic results are positive, individual study findings are highly heterogeneous, with approximately 30–40% of included studies reporting non-significant BDNF changes (Liu Y. et al., 2025). Furthermore, the BDNF Val Met polymorphism has been shown to moderate exercise-induced BDNF responses, with Met allele carriers sometimes exhibiting diminished or absent BDNF elevations following exercise (Lin et al., 2025). This genetic moderation may explain, in part, why some individuals show robust BDNF responses while others show none. The inconsistency in BDNF findings underscores the importance of methodological standardization – particularly with respect to blood sampling protocols, assay methods, and consideration of genetic factors – and cautions against overgeneralizing positive meta-analytic results to all populations and exercise protocols (Rico-González et al., 2025b). Knaepen et al.’s review systematically characterized the temporal dynamics of exercise-induced peripheral BDNF elevation – acute exercise triggers an immediate BDNF surge that subsides within 2–3 h, whereas chronic training elevates basal BDNF concentrations (Knaepen et al., 2010).

At the structural and functional brain level, chronic exercise is associated with cerebrovascular angiogenesis (via VEGF upregulation) and improves white matter microstructural integrity (Gorham and Barch, 2020; Liu Y. et al., 2025; Salvan et al., 2021). A DTI study by Chaddock-Heyman et al. revealed that children with higher aerobic fitness exhibit greater fractional anisotropy in key white matter tracts connecting prefrontal and posterior cortical regions, including the superior longitudinal fasciculus and inferior fronto-occipital fasciculus (Chaddock-Heyman et al., 2014a). A randomized controlled trial by Schaeffer et al. demonstrated that an 8-month exercise intervention significantly improved frontotemporal white matter integrity in overweight children (Schaeffer et al., 2014). Krafft et al. observed that, following an exercise intervention, prefrontal activation patterns in overweight children during executive function tasks shifted from a state of “high activation, low efficiency” toward one characterized by “precise activation, high efficiency” (Krafft et al., 2014). This pathway becomes manifest following weeks to months of sustained exercise: the initial 4–6 weeks are dominated by neurochemical adaptations (BDNF upregulation, altered neurotransmitter system sensitivity); functional connectivity reorganization begins to emerge between 6 and 12 weeks; and structural alterations in gray matter volume and cortical thickness become detectable only after 12 weeks or longer (Alvarez-Bueno et al., 2017; Donnelly et al., 2016; Mello et al., 2025; Unterguggenberger, 2025).

These two pathways are not mutually independent; rather, they are hypothesized to constitute a ‘function-to-structure’ translational continuum. According to this theoretical model, repeated acute neurochemical and hemodynamic stimuli (catecholamine release, enhanced cerebral oxygenation) may, via molecular signaling cascades (BDNF-TrkB pathway activation), accumulate into enduring neurostructural adaptations (synaptic strengthening, increased dendritic spine density, white matter myelination). However, it is important to emphasize that this translational mechanism is a theoretical proposition – it is supported by converging evidence from animal models and correlational human studies, but the direct demonstration of this causal sequence in human children remains incomplete. This model provides a useful heuristic for understanding why the effects of a single exercise bout are transient while the benefits of regular exercise may be more enduring, but it should not be mistaken for a fully validated biological cascade (Figure 2).

**FIGURE 2:**
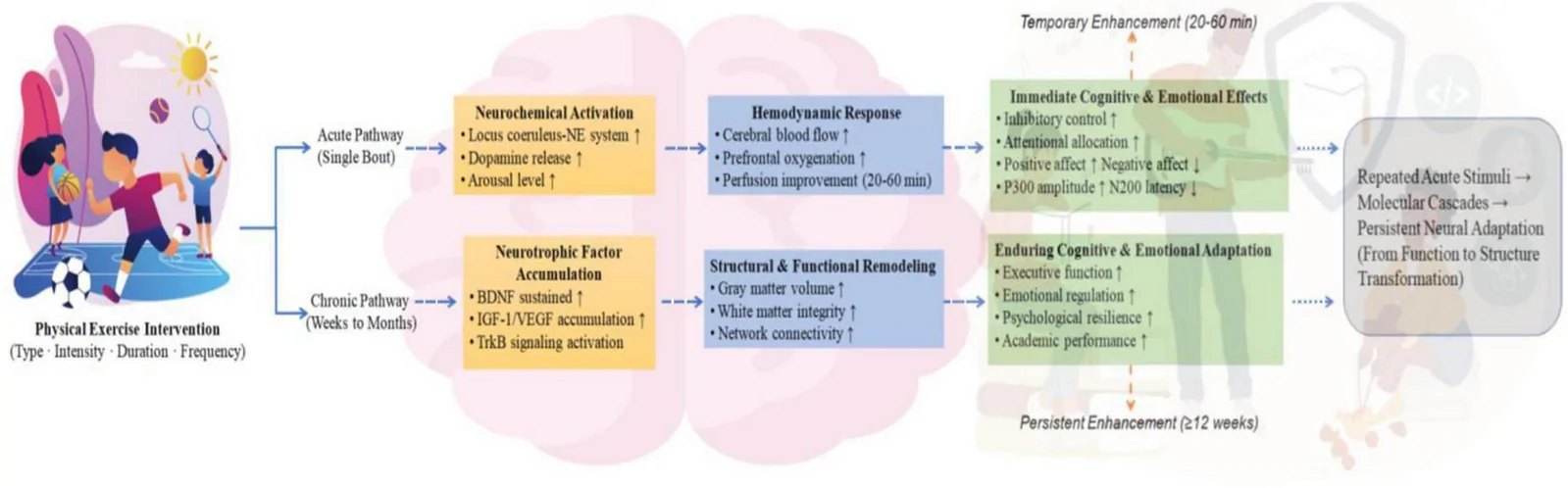
Acute and chronic pathways of exercise effects on brain function in youth. The left panel (orange-shaded region) illustrates the acute pathway following a single bout of exercise. Acute exercise triggers neurochemical activation (locus coeruleus-norepinephrine system ↑, dopamine release ↑, arousal level ↑) and hemodynamic responses (cerebral blood flow ↑, prefrontal oxygenation ↑, perfusion improvement lasting 20–60 min), leading to immediate cognitive and emotional effects including enhanced inhibitory control, improved attentional allocation, increased positive affect, and reduced negative affect. These effects are reflected electrophysiologically by increased P300 amplitude and decreased N200 latency. The right panel (blue-shaded region) depicts the chronic pathway resulting from regular exercise over weeks to months. Chronic exercise is associated with sustained upregulation of neurotrophic factors (BDNF sustained ↑, IGF-1/VEGF accumulation ↑, TrkB signaling activation) and structural/functional brain remodeling (gray matter volume ↑, white matter integrity ↑, network connectivity ↑), producing enduring cognitive and emotional adaptation characterized by stable improvements in executive function, enhanced emotional regulation, increased psychological resilience, and better academic performance. Structural changes typically emerge after ≥ 12 weeks of regular exercise. The bottom panel illustrates the hypothesized transformation mechanism linking the two pathways, according to which repeated acute neurochemical and hemodynamic stimuli may, via molecular cascades, eventually contribute to persistent neural structural adaptation. This ‘from function to structure’ process is presented as a theoretical model – a plausible account of how transient effects might accumulate – rather than as an empirically established biological sequence. Direct evidence for this translational mechanism in human pediatric populations remains limited. Visual distinction note: The arrow styles used in this Figure follow the same evidence-grading legend as Figures 3 and 4 solid arrows indicate pathways supported by convergent evidence from animal models and/or RCTs; dashed arrows indicate pathways supported primarily by correlational evidence or inferred from animal models without direct human validation. Readers should interpret the bottom panel’s “transformation mechanism” as a theoretical model (see dashed and dotted arrows in Figure 3 for comparable evidence grading). BDNF, brain-derived neurotrophic factor; IGF-1, insulin-like growth factor-1; VEGF, vascular endothelial growth factor; NE, norepinephrine. Solid arrows indicate pathways supported by convergent evidence from animal models and human RCTs; dashed arrows indicate pathways supported primarily by correlational evidence (human neuroimaging) or inferred from animal models without direct human validation.

#### 2.3.1 Interpretational note on peripheral BDNF as a surrogate marker

Before describing the molecular pathways through which exercise is hypothesized to influence brain function, it is essential to clarify a foundational methodological and interpretational issue that pervades the human literature on exercise and neuroplasticity. A substantial proportion of the evidence cited in this review derives from measurements of peripheral brain-derived neurotrophic factor (BDNF) – typically assayed from serum or plasma samples – as a proxy for central neurotrophic activity. However, this inferential link requires several critical qualifications.

*Source of peripheral BDNF.* Peripheral blood BDNF is predominantly stored in and released from platelets, with platelets containing approximately 90% of circulating BDNF. Additional sources include vascular endothelial cells, monocytes, and skeletal muscle. Peripheral BDNF concentrations thus reflect a composite of multiple tissue sources and are influenced by platelet count, platelet activation state, and circadian rhythms. Importantly, peripheral BDNF is not a direct efflux from the brain; the extent to which circulating BDNF crosses the blood-brain barrier (BBB) or correlates with brain BDNF concentrations remains controversial and incompletely characterized.

*Correspondence between peripheral and central BDNF.* The assumption that peripheral BDNF levels reflect central (brain) BDNF concentrations is primarily derived from rodent studies, some of which have reported positive correlations between serum and cortical/hippocampal BDNF levels, whereas others have found no such correspondence. In humans, the evidence is even more limited and mixed. While some studies have reported modest positive correlations between peripheral and cerebrospinal fluid (CSF) BDNF levels, these findings are inconsistent, and the correlation, when observed, is typically weak. Furthermore, CSF BDNF itself is not necessarily equivalent to brain parenchymal BDNF concentrations. No studies have directly validated the peripheral-to-central BDNF correspondence in pediatric populations, and developmental changes in BBB permeability and platelet function further complicate cross-age generalization.

*Exercise-induced changes: peripheral vs. central.* Exercise consistently elevates peripheral BDNF levels in both acute and chronic paradigms, as documented by multiple meta-analyses (see Section “2.3 Macro-level pathway model of exercise effects on brain function”). However, whether this elevation is accompanied by parallel changes in central BDNF remains largely inferred rather than demonstrated in humans. The well-established pathway from exercise to hippocampal BDNF upregulation to synaptic plasticity derives primarily from animal models (Jeon and Ha, 2017; Vaynman et al., 2004). In humans, the causal chain from exercise to peripheral BDNF elevation to brain BDNF to cognitive improvement remains a hypothesis that integrates converging lines of indirect evidence – not an empirically verified biological cascade.

*Recommendation for interpretation.* Throughout this review, we interpret peripheral BDNF findings with the following caveats: (a) peripheral BDNF is an imperfect surrogate for central BDNF and should not be equated with brain neuroplasticity; (b) the link between peripheral BDNF elevation and cognitive improvement is correlational and does not establish a BDNF-mediated causal pathway; (c) the mechanistic model linking exercise to BDNF to synaptic plasticity to executive function is a working hypothesis supported by convergent animal and human correlational evidence, not a confirmed biological sequence; and (d) direct validation of these pathways in human pediatric populations remains a critical future research priority.

To visually summarize the distinction between directly supported and inferred pathways in the BDNF-mediated exercise-brain-behavior cascade, we present Figure 3. This schematic is intended to provide readers with a clear evidence-grading framework for interpreting the mechanistic claims discussed throughout the remainder of this review.

**FIGURE 3:**
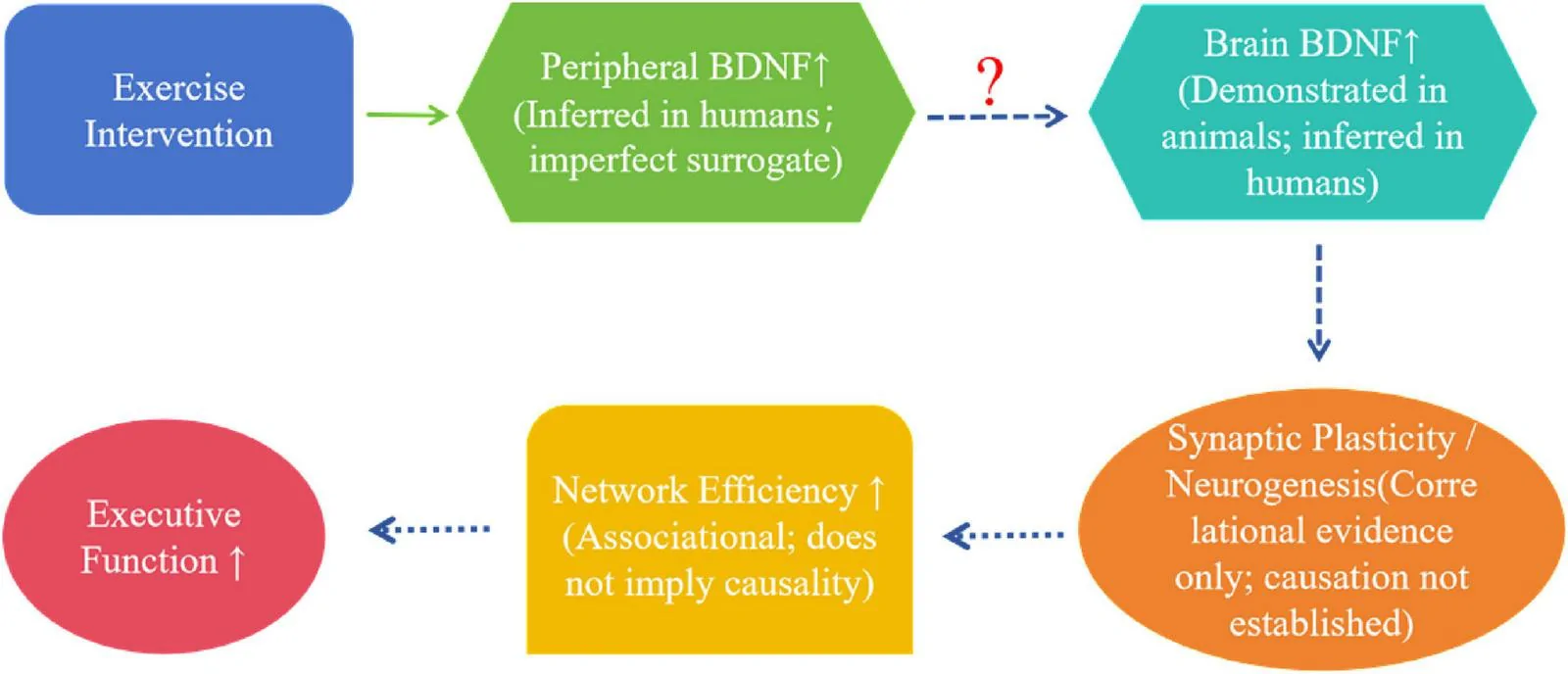
Evidence grading for the BDNF-mediated exercise-brain-behavior pathway: distinguishing direct evidence from inferred mechanisms. This schematic distinguishes direct evidence from inferred/hypothetical mechanisms in the proposed BDNF-mediated pathway from exercise to executive function. Solid arrows indicate pathways with direct empirical support: exercise-induced peripheral BDNF elevation is well-documented in human meta-analyses (Drollette et al., 2014; Hillman et al., 2009), and exercise-induced hippocampal BDNF upregulation is established in animal models (Jeon and Ha, 2017; Vaynman et al., 2004). Dashed arrows indicate pathways supported by animal model evidence but requiring cautious extrapolation to humans (e.g., BDNF → synaptic plasticity). Dotted arrows indicate pathways that are purely speculative or supported only by theoretical inference with no direct empirical evidence (e.g., synaptic plasticity → network efficiency → executive function). Most critically, the correspondence between peripheral BDNF and brain BDNF in humans is controversial and remains an imperfect surrogate relationship. This Figure is intended to clarify that the complete pathway from exercise to BDNF to network reorganization to behavioral improvement represents a conceptual synthesis of multiple lines of evidence rather than a fully validated biological cascade in youth. Red text/boxes highlight the most critical inference points requiring further investigation. Evidence-grading legend for all figures in this review: Solid arrows = established pathways with direct empirical support from human meta-analyses or RCTs; dashed arrows = inferred pathways supported by animal models or human correlational evidence; dotted arrows = speculative pathways with no direct empirical evidence. This legend applies consistently to Figures 2, 3, and 4. For detailed discussion of neuroimaging interpretational caveats (rs-FC), see Sections “2.2 Principal research paradigms and technical approaches” and “6.1.1 Interpretational limitations of resting-state functional connectivity”.

## 3 Physical exercise and executive function: effects and neural mechanisms

Before reviewing the effects of exercise on the three constituent components of executive function, an important conceptual caveat warrants explicit acknowledgment. The organization of Sections 3.1, 3.2, and 3.3 – addressing inhibitory control, working memory, and cognitive flexibility separately – follows the classic tripartite framework for heuristic clarity. However, this organizational structure should not be interpreted as a claim that these three components are fully separable neural or cognitive systems in youth, nor that exercise selectively targets them in isolation.

As discussed in Section “1.2 Definition of core concepts”, the unity versus diversity of executive function in pediatric populations remains debated. Several lines of evidence caution against strong interpretations of component-specific effects. First, the three EF components are substantially intercorrelated in developmental samples, with latent variable analyses often showing that a single common factor accounts for a large proportion of variance in EF task performance, particularly in younger children (Best and Miller, 2010; Tutakhail et al., 2026). Second, the task impurity problem means that a finding of ‘exercise improves cognitive flexibility’ based on switch costs in task-switching paradigms may reflect improvements in general task-set maintenance or working memory updating rather than flexibility-specific processes (Fernández-Rodríguez et al., 2022; Tutakhail et al., 2026). Third, neuroimaging studies in children have not consistently demonstrated the same degree of functional specialization observed in adults; prefrontal regions recruited during inhibitory control tasks also show activation during working memory and cognitive flexibility tasks, suggesting less differentiated neural substrates in developing populations (Drollette et al., 2025; Hillman et al., 2014; Segura et al., 2024).

Readers should therefore interpret the component-specific findings reviewed below with appropriate caution. The apparent differential sensitivity of inhibitory control, working memory, and cognitive flexibility to exercise interventions (as summarized in Figure 2) reflects meta-analytic patterns at the group level but does not necessarily imply that exercise selectively targets distinct neural circuits for each component. The ‘sensitivity ranking’ should be understood as a descriptive summary of the existing literature – not as evidence for the neural separability of these components in developing populations. Wherever possible, we note when effects appear to be general rather than component-specific and when task impurity limits interpretation.

### 3.1 Inhibitory control

#### 3.1.1 Behavioral level: enhancing effects of exercise interventions on inhibitory control performance

Inhibitory control is widely recognized as the component of executive function most sensitive to exercise interventions, a conclusion supported by multiple converging lines of evidence (Diamond, 2015; Diamond, 2020; González-Del-Castillo and Barbero-Alcocer, 2025). From a developmental perspective, inhibitory control is both the earliest-maturing facet of executive function and one that retains considerable plasticity throughout middle and late childhood, thereby providing a neurodevelopmental foundation for its heightened responsiveness to exercise interventions (Best and Miller, 2010).

The immediate facilitative effects of acute aerobic exercise on inhibitory control task performance have garnered relatively consistent empirical support. A meta-analysis by Chang et al. demonstrated that following a single 20–30 min bout of moderate-intensity exercise, children exhibit significantly shortened reaction times and enhanced accuracy on Flanker and Stroop tasks, with effect sizes (Hedges’ g) ranging approximately from 0.2 to 0.5 (95% CIs not reported in original analysis; small-to-moderate effects by conventional benchmarks). However, heterogeneity was moderate-to-high (I^2^ ranging from 50% to 70%), indicating substantial variation across studies in exercise protocols, participant ages, and cognitive tasks (Chang et al., 2012). A further meta-analysis by Ludyga et al. delineated the moderating roles of exercise type and intensity, revealing that coordinative exercise and open-skill sports yield superior acute effects on inhibitory control compared with closed-skill aerobic exercise (Ludyga et al., 2016). Tao et al. were among the first in the Chinese literature to systematically examine the effects of acute exercise at varying intensities on executive function, demonstrating that moderate intensity is linked to optimal facilitative effects, low intensity yields only weak effects, and high intensity may actually impair inhibitory control performance due to accumulated fatigue (Tao et al., 2025). This inverted U-shaped dose-response relationship has been corroborated by multiple subsequent investigations (Cabral et al., 2026; Şendil et al., 2026; Xu et al., 2025).

The cumulative benefits of chronic exercise interventions are similarly robust. A systematic review by Donnelly et al., encompassing 64 studies, confirmed that regular exercise sustained for at least 12 weeks exerts small-to-moderate facilitative effects on executive function in children, with inhibitory control exhibiting the most pronounced effect sizes (Donnelly et al., 2016). A meta-analysis by Álvarez-Bueno et al., focusing specifically on school-based physical education interventions, found that exercise programs integrated into the daily curriculum yield larger effect sizes for inhibitory control (*g* = 0.31) compared with extracurricular interventions (Alvarez-Bueno et al., 2017). A meta-analysis by de Greeff et al., which specifically examined the effects of exercise on executive function in preschool and school-aged children, confirmed inhibitory control as the most sensitive indicator (de Greeff et al., 2018). A meta-analysis by Singh et al., aggregating 58 randomized controlled trials, reported an overall effect size of g = 0.35 (95% CI: 0.23–0.47; a small-to-moderate effect), with subgroup analyses indicating optimal effects for interventions spanning at least 12 weeks and conducted at least three times per week (Singh et al., 2019). Xue et al., focusing on chronic exercise interventions, further corroborated the robustness of these dosage parameters (Xue et al., 2019). A recent narrative review by Guzmán-Muñoz et al., synthesizing emerging evidence from 2019 to 2025, confirmed that the sensitivity of inhibitory control to exercise exhibits cross-sample consistency across diverse cultural contexts and age cohorts (Guzmán-Muñoz et al., 2025).

Exercise type constitutes a critical factor moderating effect sizes. Cognitively engaging physical activities – that is, activities that embed cognitive challenges (e.g., strategic decision-making, rule-switching, cooperative coordination) within traditional physical exercise – demonstrate substantially greater gains in inhibitory control compared with purely repetitive aerobic exercise (Mao et al., 2024; Schmidt et al., 2016; Teng et al., 2025; Xu Y. et al., 2026). A longitudinal study by Yin et al. compared the effects of two distinct exercise intervention protocols on executive function in elementary school students, revealing that a “fancy rope-skipping” protocol incorporating cognitive challenges produced larger and more enduring benefits than a conventional running protocol (Liu et al., 2020). Xu et al. compared different types of acute moderate-intensity exercise, finding that open-skill sports such as basketball yielded superior facilitation of inhibitory control relative to closed-skill running (Xu et al., 2025). Chen et al. further demonstrated that even within the same sport (basketball), training protocols of differing intensities exert differential effects on inhibitory control (Du et al., 2025).

A recent systematic review by González-del-Castillo and Barbero-Alcocer, specifically focused on exercise interventions within school settings, confirmed that integrating physical activity into classroom instruction (e.g., between-lesson micro-breaks, movement-integrated learning) exerts significant facilitative effects on executive function – particularly inhibitory control – and, owing to its ease of implementation and low cost, possesses considerable ecological validity and translational potential (González-Del-Castillo and Barbero-Alcocer, 2025).

#### 3.1.2 Neural level: activation and reorganization of prefrontal-anterior cingulate circuitry

Neuroimaging research has elucidated the brain mechanisms underlying exercise-enhanced inhibitory control across both spatial and temporal dimensions, with core findings converging on functional enhancement and structural remodeling of prefrontal-anterior cingulate circuitry.

Following acute exercise, enhanced activation of the PFC and dorsal ACC (dACC) is observed during inhibitory tasks. Employing fNIRS, Mücke et al. demonstrated that following moderate-to-vigorous intensity exercise, children exhibit significantly elevated prefrontal oxyhemoglobin concentration during inhibitory task performance, with the magnitude of oxygenation increase significantly correlating with behavioral improvement (Mücke et al., 2018). Du et al. demonstrated that post-acute aerobic exercise improvements in Stroop task performance among university students were accompanied by increased prefrontal oxygenation, thereby providing direct functional imaging evidence for the exercise-brain-behavior association (Du et al., 2025). It is important to interpret these neuroimaging findings with caution. While statistically significant increases in prefrontal oxygenation and task-related activation are consistently observed following acute exercise, these changes represent correlates of exercise engagement rather than direct evidence of cognitive enhancement. Increased prefrontal activation does not uniquely index ‘improved neural efficiency’ – it may also reflect compensatory recruitment, increased effort, or non-neural vascular changes. Behavioral improvement remains the essential criterion for establishing functional cognitive benefit.

Event-related potential (ERP) research has meticulously characterized this effect along the temporal dimension. The foundational study by Hillman et al. revealed that children with higher cardiorespiratory fitness exhibit larger P300 amplitude and shorter P300 latency during Flanker task performance, indicative of more efficient attentional resource allocation (Hillman et al., 2009). Pontifex et al. further demonstrated that higher-fit children exhibit enhanced N200 and ERN amplitudes during incongruent trials, reflecting more efficient conflict monitoring and error detection capacities (Pontifex et al., 2011). The acute exercise study by Drollette et al. found that a single bout of moderate-intensity exercise elicited increased P300 amplitude, shortened N200 latency, and enhanced ERN amplitude during inhibitory control tasks – a neuroelectric triad that constitutes a characteristic temporal signature of acute exercise-enhanced inhibitory control (Drollette et al., 2014). González and Ludyga et al. have systematically investigated the association between exercise and electroencephalographic measures within the Chinese context, confirming the utility of P300 as a sensitive biomarker of exercise-induced cognitive benefits (González-Del-Castillo and Barbero-Alcocer, 2025; Ludyga et al., 2024).

Neural adaptations induced by chronic exercise interventions are more profound, encompassing enduring alterations at both structural and functional levels of brain organization. At the structural level, the classic investigation by Chaddock-Heyman et al. demonstrated that 9- to 10-year-old children with higher aerobic fitness exhibit greater gray matter volume in the dACC and superior frontal gyrus, with gray matter volume significantly and positively correlated with inhibitory control task performance (Chaddock-Heyman et al., 2013). Chaddock et al. further revealed a significant positive association between cardiorespiratory fitness and hippocampal volume, with hippocampal volume partially mediating the effect of exercise on memory performance (Chaddock et al., 2010). An 8-month randomized controlled trial by Krafft et al. confirmed that exercise intervention significantly increases gray matter volume in the prefrontal and cingulate cortices of overweight children (Krafft et al., 2014). It should be noted, however, that these structural associations are cross-sectional and correlational. While higher fitness is associated with greater gray matter volume, this finding does not establish that exercise caused the volumetric differences or that these differences translate into meaningful cognitive improvement. Longitudinal intervention studies with pre- and post-intervention imaging are needed to establish causal relationships between exercise-induced structural changes and behavioral outcomes. A recent study by Brower et al. demonstrated that the duration of sports participation during childhood and adolescence is positively correlated with dorsolateral prefrontal cortical thickness, providing developmental evidence for exercise-induced structural brain plasticity (Brower et al., 2025).

At the functional level, Voss et al., employing resting-state fMRI (rs-fMRI), demonstrated that children with higher aerobic fitness exhibit stronger within-network functional connectivity in cognitive control-related networks – including the frontoparietal control network and cingulo-opercular network – with network efficiency positively correlated with inhibitory control performance (Voss et al., 2011). Task-based fMRI by Chaddock-Heyman et al. revealed that higher-fit children exhibit more focal activation patterns in prefrontal and parietal regions during inhibitory tasks, characterized by “efficient” activation – enhanced activation in task-relevant regions coupled with more complete deactivation in task-irrelevant regions (Chaddock-Heyman et al., 2013). Intervention research by Krafft et al. found that following 8 months of exercise, prefrontal activation in overweight children during inhibitory tasks transitioned from a diffuse, widespread pattern pre-intervention to a precise, focal pattern post-intervention, with this shift in activation pattern occurring concomitantly with behavioral improvement (Krafft et al., 2014).

Notable findings derive from a recent study by Cline et al.. Employing a crossover design, this investigation examined the effects of a single 20-min bout of moderate-intensity exercise on rs-FC in children aged 9–13 years. Results revealed that exercise significantly enhanced within-network functional connectivity of the frontoparietal network and strengthened anti-correlation between the frontoparietal network and the DMN. This finding indicates that acute exercise is associated with altered rs-FC, specifically enhanced within-network frontoparietal network (FPN) connectivity and stronger FPN-DMN anti-correlation. While the authors of the original study interpreted this pattern as potential evidence of improved network segregation, these connectivity changes are correlational (see Sections “2.2 Principal research paradigms and technical approaches” and “6.1.1 Interpretational limitations of resting-state functional connectivity”), and alternative interpretations – including compensatory recruitment, reduced inhibitory tone, or physiological artifacts – remain equally plausible (Cline et al., 2024).

The FITKids randomized controlled trial by Hillman et al. represents a landmark investigation in this domain. This study randomized 221 children aged 8–9 years to either a 9-month after-school exercise intervention or a waitlist control group, demonstrating that children in the intervention group exhibited significant improvements in inhibitory control behavioral performance, accompanied by increased P300 amplitude and enhanced prefrontal activation efficiency (Hillman et al., 2014). A randomized controlled trial by Davis et al. corroborated similar effects in overweight children – following a 13-week exercise intervention, the intervention group exhibited improved inhibitory control and enhanced prefrontal activation. These two well-conducted RCTs provide some of the strongest available evidence for a causal effect of exercise on inhibitory control. However, it should be noted that these studies represent single independent trials with specific populations (overweight children in the Davis et al. study; typically developing 8- to 9-year-olds in the FITKids study), and their findings require replication in diverse populations before they can be considered generalizable causal evidence. Taken together with the broader meta-analytic evidence, these RCTs support the conclusion that exercise interventions can improve inhibitory control, although the strength of this evidence is best characterized as Moderate (see Table 2) rather than conclusive (Davis et al., 2011).

Synthesizing the aforementioned evidence, prefrontal-anterior cingulate circuitry constitutes the core neural substrate underlying exercise-enhanced inhibitory control (Evidence tier: Human neuroimaging (correlational) + animal models (mechanistic); structural/functional associations not causal; Low to Moderate confidence; see Table 2). Acute exercise is linked to immediate functional enhancement by augmenting metabolic support and neurotransmitter availability within this circuitry, whereas chronic exercise engenders enduring neural adaptations through the induction of increased gray matter volume, improved white matter integrity, and reorganization of functional connectivity. This temporal sequence – characterized by “function first, structure follows” – reflects the translational logic of neuroplasticity from transient to stable states.

Evidence demarcation: The behavioral findings in Section “3.1.1 Behavioral level: enhancing effects of exercise interventions on inhibitory control performance” are supported by causal evidence from RCTs. The neuroimaging findings in this section are correlational (see Sections “2.2 Principal research paradigms and technical approaches” and “6.1.1 Interpretational limitations of resting-state functional connectivity”). The temporal sequence of ‘function first, structure follows’ remains a theoretical interpretation.

### 3.2 Working memory

#### 3.2.1 Behavioral level: effects of exercise on working memory updating and maintenance

The response pattern of working memory to exercise interventions is more complex than that observed for inhibitory control, with overall effect sizes being comparatively smaller and more pronouncedly moderated by exercise type and cognitive load (Gonçalves et al., 2025; Kjellenberg et al., 2024; Singh et al., 2019).

The optimal effects of moderate-intensity aerobic exercise on N-back task performance have garnered support from the majority of studies; however, considerable heterogeneity exists in the magnitude of reported effect sizes. A randomized controlled trial by Kamijo et al. demonstrated that a 9-month after-school exercise intervention significantly improved working memory performance in children aged 9–10 years, with effects persisting beyond the intervention period (Kamijo et al., 2011). A meta-analysis by de Greeff et al. revealed that the effect size of exercise on working memory (g = 0.24; 95% CI not reported in the meta-analysis; a small effect) is smaller than that for inhibitory control (g = 0.35; small-to-moderate). Heterogeneity for working memory outcomes was higher (I^2^ ≈ 55–60%) than for inhibitory control (I^2^ ≈ 40–45%), suggesting greater variability in working memory findings across studies (de Greeff et al., 2018).

It is important to acknowledge that not all studies have found significant exercise effects on working memory. Several randomized controlled trials have reported null or non-significant findings for working memory outcomes, even when inhibitory control showed significant improvements within the same sample (Kamijo et al., 2011). For example, the seminal FITKids trial by Hillman et al. (2014) found that while inhibitory control improved significantly, working memory effects were more modest and did not reach statistical significance in some analyses (Hillman et al., 2014). Similarly, a study by Kamijo et al. (2011) reported that exercise-induced working memory benefits were task-load dependent, with significant improvements observed on 1-back but not 3-back or 2-back tasks (Kamijo et al., 2011). This load-dependent pattern suggests that working memory benefits may be limited to less demanding tasks or may require greater intervention intensity to manifest on complex tasks.

The heterogeneity in working memory findings is reflected in the higher I^2^ values (≈55–60%) compared with inhibitory control (≈40–45%), indicating greater variability across studies. Several factors may account for this inconsistency (Kou et al., 2024). First, working memory engages a more diffuse prefrontal-parietal-hippocampal network than inhibitory control, and functional reorganization of this distributed network may require longer intervention durations or greater cumulative stimulus dosage. Second, the diversity of working memory tasks used across studies (e.g., N-back, digit span, spatial working memory) may tap different cognitive and neural processes, complicating cross-study comparison. Third, studies with smaller sample sizes may be underpowered to detect the smaller effect sizes characteristic of working memory outcomes (g ≈ 0.24 vs. g ≈ 0.35 for inhibitory control). Readers should therefore interpret the positive meta-analytic estimate for working memory with caution, recognizing that null findings are common and that the evidence base is less consistent than for inhibitory control.”

The influence of exercise type on working memory is particularly salient. Coordinative exercise (e.g., rope skipping, gymnastics) and open-skill sports (e.g., ball games) confer more pronounced benefits for working memory compared with closed-skill aerobic exercise (e.g., stationary cycling) (Singh et al., 2026; Tomporowski et al., 2015). Tomporowski et al., in a theoretical analysis, posited that coordinative exercise demands the planning, monitoring, and dynamic adjustment of movement sequences – cognitive operations that directly recruit the working memory system – thereby constituting a form of “cognitive-motor synergistic training” (Tomporowski et al., 2015). A network meta-analysis comparing the intervention effects of diverse exercise modalities on the mental health of youth found that coordinative exercise and team sports were superior to isolated aerobic exercise in conferring cognitive benefits (Singh et al., 2026).

The differentiation between acute and chronic effects also warrants attention. The facilitative effect of acute exercise on working memory exhibits an inverted U-shaped temporal profile – peaking approximately 20–30 min post-exercise and gradually returning to baseline after approximately 60 min (Chang et al., 2012; Ludyga et al., 2016). In contrast, chronic exercise yields more enduring benefits through structural and functional remodeling of hippocampal-prefrontal circuitry, with effect sizes accumulating as the intervention period extends (Donnelly et al., 2016; Huang et al., 2014).

#### 3.2.2 Neural level: efficiency optimization of the dorsolateral prefrontal-parietal network

The core neural substrate of working memory is the FPN, comprising the dlPFC and posterior parietal cortex. At the neural level, the facilitative effects of exercise interventions on working memory are manifested as enhanced activation efficiency and strengthened functional connectivity within this network.

At the structural level, Chaddock et al. demonstrated that children with higher cardiorespiratory fitness possess larger hippocampal volume, with hippocampal volume exhibiting a significant positive correlation with relational memory performance. This finding provides crucial structural imaging evidence for the exercise-hippocampus-memory causal continuum (Chaddock et al., 2010). A DTI study by Schaeffer et al. further revealed that an 8-month exercise intervention significantly improved the microstructural integrity of white matter tracts connecting prefrontal and temporal cortices in overweight children, with the magnitude of improvement correlating with enhanced working memory performance (Schaeffer et al., 2014).

At the functional level, task-based fMRI by Chaddock-Heyman et al. demonstrated that higher-fit children exhibit more focal activation of the dlPFC during N-back task performance, coupled with more complete deactivation of task-irrelevant brain regions (Chaddock-Heyman et al., 2013). Notably, this positive correlation does not establish that enhanced connectivity mediates or causes the working memory advantage – alternative explanations, such as shared effects of fitness on both neural and cognitive measures, or the influence of unmeasured confounders (e.g., socioeconomic status, baseline cognitive ability), cannot be ruled out (Voss et al., 2011). Consistent with this caution, the neuroimaging findings reviewed in this section should be interpreted as correlates of fitness or exercise engagement rather than as direct evidence of causation (see Sections “2.2 Principal research paradigms and technical approaches” and “6.1.1 Interpretational limitations of resting-state functional connectivity”). Behavioral validation remains essential for any functional interpretation. In the studies cited above, behavioral improvements were generally reported alongside neuroimaging changes, but the correspondence between the two was often modest and the studies were not designed to test mediation.

Hippocampal-prefrontal functional coupling plays a pivotal role in working memory updating. Evidence from animal models indicates that exercise-induced improvements in hippocampal neurogenesis and synaptic plasticity are contingent upon activation of the BDNF-TrkB signaling pathway (Knaepen et al., 2010; Vaynman et al., 2004). The seminal work by Vaynman et al. demonstrated that exercise-induced BDNF upregulation promotes hippocampal long-term potentiation (LTP) – the cellular mechanism underlying learning and memory – through activation of the MAPK/ERK and PI3K/Akt signaling cascades (Vaynman et al., 2004). Although human research is constrained by the infeasibility of invasive assays, the positive correlation between peripheral BDNF levels and working memory performance provides indirect support for the cross-species conservation of this mechanistic pathway (Dinoff et al., 2016; Huang et al., 2014; Jeon and Ha, 2017; Rico-González et al., 2025a; Szuhany et al., 2015). Specifically, an adolescent study reported a significant positive association between the magnitude of high-intensity exercise-induced BDNF elevation and the corresponding degree of improvement in working memory task performance.

It is important to note that the neural substrate of working memory is more diffuse than that of inhibitory control, encompassing coordinated engagement of a more extensive prefrontal-parietal-hippocampal network. This may account for why the responsiveness of working memory to exercise interventions is comparatively more gradual and characterized by smaller effect sizes – functional reorganization of a distributed network necessitates longer intervention durations and greater cumulative stimulus dosage to manifest as observable behavioral change.

Evidence demarcation: The working memory findings combine behavioral RCTs (causal at the behavioral level) with correlational neuroimaging associations. The inference that network reorganization mediates improvement has not been directly tested (see Sections “2.2 Principal research paradigms and technical approaches” and “6.1.1 Interpretational limitations of resting-state functional connectivity”).

### 3.3 Cognitive flexibility

#### 3.3.1 Behavioral level: facilitation of task switching and set-shifting capacity through exercise

Cognitive flexibility represents the latest-maturing and most hierarchically advanced component of executive function, and its responsiveness to exercise interventions exhibits a distinctive dependence on exercise modality (Diamond, 2020; Wang et al., 2023; Wang et al., 2026).

Team sports and open-skill sports confer significantly greater benefits for cognitive flexibility compared with repetitive, closed-skill exercise. Research by Pesce et al. demonstrated that adolescents participating in team sports exhibit significantly smaller switch costs in task-switching paradigms relative to their peers engaged in individual sports. This finding aligns with the principle of “training specificity”: the inherent dynamic unpredictability of team sport environments naturally compels participants to engage in continuous attentional shifting, strategic adjustment, and behavioral adaptation, thereby constituting direct training of cognitive flexibility (Pesce et al., 2016). A meta-analysis by Vazou et al. further confirmed that cognitively engaging physical activities yield a larger effect size for cognitive flexibility (g = 0.42; a small-to-moderate effect) compared with traditional aerobic exercise (g = 0.28; a small effect). However, heterogeneity was moderate (I^2^ ≈ 50%), and the authors noted that few studies directly compared these modalities within the same design (Vazou et al., 2019).

The relationship between exercise intensity and cognitive flexibility conforms to an inverted U-shaped profile: moderate intensity is linked to optimal facilitative effects, low intensity yields only weak effects, and high intensity may transiently impair flexibility performance due to accumulated fatigue and depletion of cognitive resources (Li et al., 2025c; Xu et al., 2025). Li et al. found that switch costs in cognitive flexibility tasks actually increased following high-intensity exercise, suggesting that exercise prescriptions targeting cognitive flexibility necessitate particularly precise calibration of intensity (Li et al., 2025c). A longitudinal study by Xu et al. further demonstrated that exercise protocols incorporating cognitive challenges yield superior cumulative benefits for cognitive flexibility compared with purely aerobic exercise, with effects persisting for a longer duration following intervention cessation (Xu et al., 2025).

As a critical mediating variable in the transfer of exercise-related gains to academic adaptation, the educational significance of cognitive flexibility warrants considerable attention. A systematic review by Donnelly et al. revealed that improvements in cognitive flexibility serve a significant mediating role in the facilitative effects of exercise interventions on academic achievement, with effects being particularly pronounced for academic tasks requiring flexible switching among multiple strategies – such as mathematical problem-solving and reading comprehension (Donnelly et al., 2016). A school-based intervention study by Liu et al. corroborated this mediational pathway (Liu et al., 2020).

#### 3.3.2 Neural level: synergistic reorganization of Fronto-Parieto-Striatal circuitry

The neural substrate of cognitive flexibility involves the coordinated operation of the frontopolar cortex, dlPFC, posterior parietal cortex, and dorsal striatum – constituting a more complex neural circuit than those subserving inhibitory control and working memory (Hillman et al., 2014; Lang et al., 2025).

The FITKids trial by Hillman et al. revealed that following a 9-month exercise intervention, children exhibited enhanced activation of the frontopolar cortex and dorsal striatum during task-switching performance, with the magnitude of activation increase significantly correlated with reductions in switch cost. This finding suggests that exercise may optimize cognitive flexibility by enhancing the functional integrity of frontopolar-striatal circuitry (Hillman et al., 2014). A recent study by Lang et al. provided crucial evidence from a developmental perspective. Leveraging data from the Human Connectome Project-Development (HCP-D), this investigation demonstrated that adolescents with a history of sports participation exhibited significantly superior performance on an emotional face recognition task compared with their non-sport-participating peers, with advantages evident across the recognition speed of fearful, happy, and sad facial expressions. Moreover, sports participation history was positively correlated with gray matter volume in social cognition-related brain regions, including the superior temporal sulcus and fusiform face area, suggesting that exercise-induced facilitation of social cognitive flexibility may engage a more extensive temporal-prefrontal network (Lang et al., 2025).

The role of the striatum in cognitive flexibility merits particular attention. As a critical hub for habit formation and behavioral switching, the dorsal striatum receives cognitive control signals from the PFC and modulates the selection and switching of behavioral strategies via dopaminergic afferents. Exercise-induced dopamine release may optimize cognitive flexibility by enhancing the signal-to-noise ratio within the striatum. It is important to emphasize that the interpretation of these neuroimaging findings – particularly the inference that reduced switch cost reflects ‘optimized’ cognitive flexibility – is based on indirect evidence. Alternative interpretations remain plausible (see Sections “2.2 Principal research paradigms and technical approaches” and “6.1.1 Interpretational limitations of resting-state functional connectivity”), and without behavioral validation, these findings should be considered suggestive rather than definitive. Diamond noted that cognitive flexibility is the executive function component most sensitive to exercise modality, and the unique advantages conferred by open-skill sports and cognitively engaging physical activities stem precisely from their direct recruitment of frontopolar-striatal circuitry (Diamond, 2015).

Evidence demarcation: The neural findings for cognitive flexibility are predominantly correlational. While the FITKids trial provides behavioral-level causal evidence, the interpretation that frontopolar-striatal activation patterns ‘optimize’ cognitive flexibility is inferred from correlational neuroimaging and requires behavioral validation (see Sections Sections “2.2 Principal research paradigms and technical approaches” and “6.1.1 Interpretational limitations of resting-state functional connectivity”).

### 3.4 An integrative neural mechanism model of executive function enhancement

The tripartite framework described below is a conceptual model integrating evidence from animal studies, human correlational neuroimaging, and behavioral interventions. While each component pathway is empirically supported (Table 4), the causal sequence linking them remains inferred rather than established in human pediatric populations (see Section “2.3 Macro-level pathway model of exercise effects on brain function” and Figure 3). We present this model as a generative framework for guiding future research, not as a definitive account of causal mechanisms.

It is important to emphasize three additional caveats. First, the three pathways – molecular, hemodynamic, and network – are not equally supported by evidence; their presentation as an integrated framework is a heuristic device for organizing existing findings, not a claim that they operate as a unified sequence in the brain. Second, the vertical arrangement of the pathways in Figure 2 (molecular at top, network at bottom) is diagrammatic and does not imply a fixed temporal or causal ordering; the pathways likely operate in parallel and interact in complex ways that are not yet fully understood. Third, the proposed sequence from BDNF to synaptic plasticity to network efficiency to executive function is a theoretical synthesis – plausible and useful, but requiring direct experimental validation in human pediatric populations. Synthesizing findings across the three constituent components of executive function, the neural mechanisms through which exercise may enhance executive function can be distilled into three interconnected core pathways, constituting an integrative tripartite framework encompassing “molecular, hemodynamic, and network” levels of analysis (Li et al., 2025a; Stillman et al., 2020).

The Neurochemical Pathway is centered on brain-derived neurotrophic factor (BDNF) as a hypothesized pivotal hub. The evidence status of each step in this proposed cascade is explicitly distinguished in Table 4 and discussed in detail in Section “2.3.1 Interpretational note on peripheral BDNF as a surrogate marker” (peripheral-central BDNF correspondence) and Figure 3 (evidence grading). In brief: (a) Exercise-peripheral BDNF elevation is well-established in human meta-analyses and RCTs (Evidence: High; direct human measures) (Dinoff et al., 2016; Szuhany et al., 2015). (b) Exercise-central BDNF upregulation is directly demonstrated in animal models but remains inferred in humans, as peripheral BDNF is an imperfect surrogate for brain BDNF (Evidence: Very Low for human central BDNF; see Section “2.3.1 Interpretational note on peripheral BDNF as a surrogate marker” and Figure 3) (Knaepen et al., 2010; Vaynman et al., 2004). (c) BDNF-TrkB-MAPK/ERK/PI3K/Akt signaling is well-established in animal models (Evidence: Low for human inference); (d) BDNF-synaptic plasticity is directly demonstrated in rodents (Evidence: Low for human inference); (e) synaptic plasticity-network efficiency is purely theoretical (Evidence: Very Low; speculative); and (f) network efficiency-executive function is correlational (Evidence: Low; correlational only). Readers are referred to Table 4 and Section “2.3.1 Interpretational note on peripheral BDNF as a surrogate marker” for detailed evidence grading.

In animal models, exercise-induced muscle contraction has been shown to trigger the release of fibronectin type III domain-containing protein 5 (FNDC5)/irisin, which is hypothesized to cross the blood-brain barrier and activate BDNF transcription and translation within the rodent brain (Knaepen et al., 2010; Vaynman et al., 2004). However, this pathway remains largely inferred in humans, as direct evidence for FNDC5/irisin crossing the human BBB or for peripheral BDNF elevation reflecting central BDNF changes is currently lacking. Upon binding to TrkB receptors, BDNF may activate downstream signaling cascades, thereby promoting synaptic plasticity. Crucially, readers should not interpret the complete sequence from BDNF to synaptic plasticity to network efficiency to executive function as a fully established causal chain (Dinoff et al., 2016; Huang et al., 2014; Jeon and Ha, 2017; Knaepen et al., 2010; Rico-González et al., 2025a; Szuhany et al., 2015; Vaynman et al., 2004). The evidence supporting this cascade is strongest at its beginning (exercise → peripheral BDNF) and weakest at its end (network efficiency → executive function), with intermediate steps supported primarily by animal models and theoretical inference (Knaepen et al., 2010).

The Cerebrovascular Pathway involves exercise-induced upregulation of VEGF and improved cerebral blood flow perfusion. Acute exercise may enhance prefrontal oxygenation by increasing cardiac output and cerebrovascular reactivity to carbon dioxide, with fNIRS monitoring confirming that elevated prefrontal oxyhemoglobin concentration persists for 20–40 min post-exercise (Du et al., 2025; Mücke et al., 2018). Chronic exercise, in contrast, augments basal perfusion levels of brain tissue by promoting capillary neogenesis (angiogenesis) and vascular remodeling, thereby providing more robust metabolic support for neural activity (Chaddock-Heyman et al., 2014a; Krafft et al., 2014; Schaeffer et al., 2014). Improved cerebral perfusion not only supplies the energetic substrate for acute cognitive enhancement but also indirectly supports neuroplasticity by facilitating the transport and distribution of neurotrophic factors.

The Neural Network Pathway describes the functional reorganization of large-scale brain networks. Exercise interventions are associated with alterations in within-network functional connectivity of the frontoparietal control network and in the coupling between the DMN and task-positive networks (Chaddock-Heyman et al., 2014a; Cline et al., 2024; Voss et al., 2011). These network-level associations have been interpreted in the literature as potential correlates of cognitive control optimization, but this interpretation remains correlational and should not be mistaken for a mechanistic explanation. The causal role of rs-FC changes in mediating behavioral improvement has not been established (Cline et al., 2024). Longitudinal studies indicate that improvements in aerobic fitness are associated with attenuated within-network connectivity of the DMN and enhanced within-network connectivity of the FPN – a pattern of network reconfiguration that aligns closely with behavioral improvements in executive function (Hillman et al., 2014; Voss et al., 2011). Importantly, these network-level associations are derived from correlational rs-FC studies and should not be interpreted as evidence of causal mechanism (see Section “6.1.1 Interpretational limitations of resting-state functional connectivity”). Without corresponding behavioral improvements, network-level changes remain correlates rather than evidence of functional benefit.

Within this conceptual model, the three pathways are hypothesized to interact in a coordinated manner: BDNF upregulation and improved cerebral perfusion may support synaptic plasticity, which in turn may enable the functional reorganization of large-scale networks. However, it is important to emphasize that this hierarchical relationship is a theoretical proposition, not an empirically established sequence. The causal links between molecular changes, hemodynamic adaptations, and network reorganization have not been directly tested in human pediatric populations, and alternative models – in which these pathways operate independently, in parallel, or with different temporal dynamics – remain equally plausible. This integrative framework of “molecular priming – hemodynamic support – network expression” provides a conceptual heuristic for organizing existing findings and generating testable hypotheses about the multi-level pathways through which exercise may influence executive function. This model should be understood as a synthesis of converging correlational and experimental evidence, not as a definitive account of the causal mechanisms. It also accounts for why the effect patterns of different intervention protocols (acute versus chronic, aerobic versus cognitively engaging) exhibit both convergence and divergence – they may differentially weight the activation of these three pathways to produce specific cognitive benefits. Readers are encouraged to consult Table 4 for a detailed evidence grading of each individual pathway and to interpret the integrated framework in light of the varying levels of support across its components. As shown in Figure 4, the three pathways are depicted with different arrow styles (solid, dashed, and dotted) to indicate the strength of evidence supporting each link. Readers are encouraged to consult the figure’s legend and Table 4 when interpreting the evidential status of each proposed mechanism.

**FIGURE 4:**
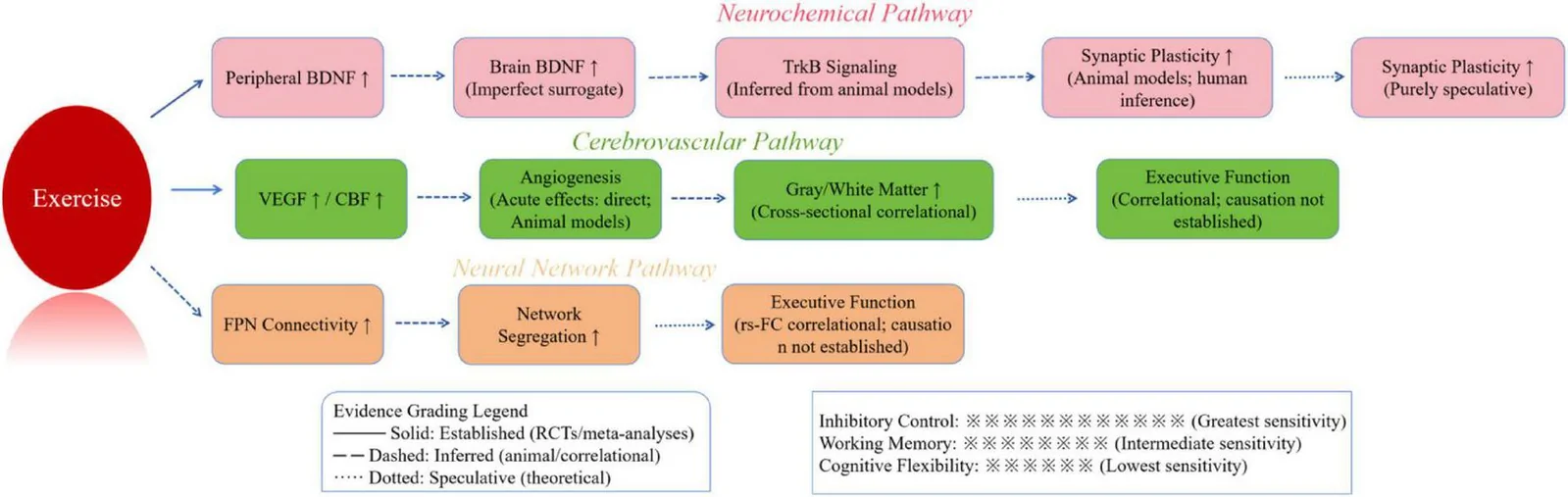
The ‘molecular-hemodynamic-network’ triad model of exercise-enhanced executive function in youth – a conceptual framework with evidence grading. This Figure presents an integrative conceptual model comprising three pathways hypothesized to mediate the effects of exercise on executive function. Crucially, this Figure is a schematic representation of a theoretical model, not a diagram of an empirically established biological sequence. The vertical arrangement of the pathways is diagrammatic and does not imply a fixed temporal or causal ordering; the pathways likely interact in complex, parallel, and dynamic ways that are not yet fully characterized. Arrow styles indicate the strength of evidence supporting each link: solid arrows indicate pathways with direct empirical support from human meta-analyses or RCTs; dashed arrows indicate pathways supported by animal model evidence or human correlational studies but requiring cautious interpretation; dotted arrows indicate pathways that are purely speculative or supported only by theoretical inference (see legend in Figure). The evidence supporting each pathway varies considerably (see Table 3), and the model as a whole should be interpreted as a heuristic for organizing findings and generating hypotheses rather than as a definitive account of causal mechanisms. Upper panel: Neurochemical pathway (molecular level). In animal models, exercise upregulates BDNF, IGF-1, dopamine, and norepinephrine, which activate downstream signaling cascades (MAPK/ERK and PI3K/Akt) to promote synaptic plasticity, hippocampal neurogenesis, and increased dendritic spine density. The translation of these molecular events to human cognitive benefits remains largely inferred. Middle panel: Cerebrovascular pathway (hemodynamic level). Exercise elevates VEGF expression, increases cerebral blood flow and prefrontal oxygenation, and promotes capillary angiogenesis. Acute effects manifest as immediate perfusion increases (well-supported), while chronic adaptations including elevated baseline perfusion and enhanced cerebrovascular reserve, and their link to executive function improvement, are supported by correlational evidence. Bottom panel: Neural network pathway (systems level). Correlational neuroimaging studies show that exercise is associated with enhanced intra-network connectivity within the frontoparietal control network, strengthened anti-correlation between the default mode network (DMN, a set of brain regions active during rest and mind-wandering) and task-positive networks, and optimized network segregation. The causal role of these network changes in driving behavioral improvement remains to be established. Evidence note: Solid arrows indicate pathways supported by convergent evidence from animal models and/or randomized controlled trials. Dashed arrows indicate pathways supported primarily by correlational evidence (human neuroimaging) or inferred from animal models without direct human validation; these represent hypothesized links requiring further investigation. Dotted arrows indicate pathways that are purely speculative or supported only by theoretical inference with no direct empirical evidence. The bottom section displays the observed differential sensitivity of the three executive function components to exercise intervention based on meta-analytic findings, with inhibitory control (↑) showing the largest effect sizes in the current literature, followed by working memory (↑) and cognitive flexibility (↑). It is important to emphasize that this ranking reflects group-level meta-analytic patterns and should not be interpreted as evidence that the three components are fully separable neural systems in children, nor that exercise selectively targets distinct neural circuits for each component. Alternative explanations – including task impurity (different tasks recruit multiple cognitive processes), developmental measurement invariance, and the greater frequency of inhibitory control tasks in intervention studies – may partially account for the observed differences in effect sizes. Interpretational note on rs-FC findings: The network-level pathway in this Figure is derived from rs-FC studies, which are inherently correlational (see Sections “2.2 Principal research paradigms and technical approaches” and “6.1.1 Interpretational limitations of resting-state functional connectivity”). Changes in rs-FC do not directly measure ‘neural efficiency’ or ‘cognitive control,’ and should be interpreted as correlates of exercise engagement rather than established mechanisms. BDNF, brain-derived neurotrophic factor; IGF-1, insulin-like growth factor-1; VEGF, vascular endothelial growth factor; DMN, default mode network; MAPK, mitogen-activated protein kinase; ERK, extracellular signal-regulated kinase; PI3K, phosphoinositide 3-kinase; CBF, cerebral blood flow; FPN, frontoparietal network.

## 4 Physical exercise and emotional brain regions: effects and neural mechanisms

### 4.1 Behavioral evidence for exercise-induced improvements in emotional health

#### 4.1.1 Cultivation of positive affect and buffering of negative affect

The facilitative effects of regular physical exercise on the emotional health of youth have garnered substantial support from behavioral evidence. Although effect sizes fall within the small-to-moderate range, the public health significance of exercise interventions is considerable given their low cost, minimal risk, and high accessibility (Ahn and Fedewa, 2011; Biddle et al., 2019; Lubans et al., 2016).

An updated umbrella review by Biddle et al. systematically evaluated the strength of evidence concerning the relationship between physical activity and mental health in youth. Encompassing 42 systematic reviews, the review concluded that exercise exerts significant alleviating effects on depressive symptoms (effect sizes approximately -0.4 to -0.6; small-to-moderate effects on standardized mean difference scales) and anxiety levels (effect sizes approximately -0.3 to -0.5; small-to-moderate effects), with the strength of evidence rated as moderate to strong (Biddle et al., 2019). A mechanistic review by Lubans et al. further delineated six potential pathways through which exercise improves adolescent mental health: neurobiological pathways (BDNF, endocannabinoid system), psychosocial pathways (self-efficacy, social support, sense of mastery), and behavioral pathways (improved sleep, reduced screen time), noting that synergistic interactions among multiple pathways are critical to the emotional benefits conferred by exercise (Lubans et al., 2016).

A meta-analysis by Ahn and Fedewa, aggregating 73 studies, confirmed an overall effect size of physical activity on children’s mental health of 0.30 (95% CI: 0.19–0.41; a small-to-moderate effect), with effects on self-esteem enhancement (0.35; small-to-moderate) exceeding those on anxiety reduction (0.28; small) (Ahn and Fedewa, 2011). A systematic review by Brown et al., focusing specifically on the intervention effects of exercise on adolescent depression, found that exercise intervention groups exhibited significantly greater improvement in depressive symptoms compared with non-exercise control groups, with effects evident in both clinical and subclinical populations (Brown et al., 2013). A meta-analysis by Carter et al., which specifically examined the effects of exercise on depressive symptoms in adolescents, included 11 randomized controlled trials and reported a moderate overall effect size (SMD = -0.48; 95% CI: -0.66 to -0.30; a moderate effect). Heterogeneity was moderate (I^2^ ≈ 55%) (Carter et al., 2016). A meta-analysis by Bailey et al., encompassing 17 RCTs, confirmed a significant effect of exercise on alleviating adolescent depression (SMD = -0.62; 95% CI: -0.82 to -0.42; a moderate-to-large effect), with effect sizes increasing as intervention duration extended. Heterogeneity was moderate (I^2^ ≈ 50%) (Bailey et al., 2018).

The immediate emotional benefits of acute exercise are similarly robust. A meta-analysis by Li et al., comprising 24 studies, found that physical activity interventions exerted a significant overall effect on positive affect in youth (SMD = 0.62), with subgroup analyses indicating optimal effects for 30–60 min of aerobic exercise among adolescents aged ≥ 12 years, and effects persisting for 2–4 h post-exercise (Li et al., 2022). A systematic review by Rodriguez-Ayllon et al., encompassing 114 studies, comprehensively assessed the impact of physical activity and sedentary behavior on the mental health of youth, with conclusions strongly supporting the protective role of moderate-to-vigorous physical activity (Rodriguez-Ayllon et al., 2019). A recent meta-analysis by Wang et al., aggregating 29 RCTs, confirmed significant effects of exercise on alleviating both adolescent depression (*g* = -0.57) and anxiety (*g* = -0.41) (Zhou et al., 2025).

Domestic research has likewise contributed substantial evidence within the Chinese context. Guzmán-Muñoz et al. reviewed the effects of physical exercise on emotion regulation capacity in adolescents, noting that exercise improves emotion regulation by enhancing prefrontal-amygdala functional connectivity (Guzmán-Muñoz et al., 2025). Zhou et al. examined the effects of acute aerobic exercise on emotion regulation, finding that moderate-intensity exercise elevates positive affect and reduces negative affect, with effects most pronounced immediately post-exercise (Zhou et al., 2025). A meta-analysis by Cortese et al., aggregating 28 studies, confirmed a significant alleviating effect of exercise interventions on negative affect in adolescents (SMD = -0.52) (Singh et al., 2026). Singh et al., systematically reviewed the neural mechanisms through which exercise influences emotional health (Singh et al., 2025). Ooms et al. synthesized the relationship between physical exercise and mental health across cognitive, emotional, and brain mechanistic levels (Ooms et al., 2025).

Regarding socioemotional benefits, a systematic review by Eime et al., comprising 30 studies, confirmed the multidimensional benefits of sports participation for the psychological and social health of youth, including enhanced self-esteem, improved social skills, and reduced social anxiety (Eime et al., 2013). A longitudinal study by Vella et al. found that childhood sports participation significantly predicted lower levels of psychological difficulties two years later, independent of baseline mental health status (Vella et al., 2015). Vella et al. further identified a bidirectional facilitative relationship between sports participation and adolescent mental health – adolescents with better mental health are more likely to sustain sports participation, while sports participation in turn further may enhance mental health (Vella et al., 2017). Tang et al. (Teng et al., 2025) demonstrated that physical exercise promotes adolescent social adaptation by enhancing psychological capital and emotion regulation capacity (Tang et al., 2025).

#### 4.1.2 Effects of exercise interventions on emotion regulation capacity

Emotion regulation capacity – defined as the ability to monitor, evaluate, and modulate emotional responses in the service of goal attainment – constitutes a critical mediating variable through which exercise influences psychological adaptation. Longitudinal studies have demonstrated that adolescents who engage in regular physical activity score significantly higher on adaptive regulation strategies, including emotional awareness, emotional expression, and cognitive reappraisal, while scoring significantly lower on maladaptive strategies such as expressive suppression, compared with their sedentary counterparts (Brown et al., 2013; Guzmán-Muñoz et al., 2025).

Randomized controlled trials conducted within school physical education settings have further corroborated that physical education intervention programs incorporating emotional themes significantly enhance students’ self-efficacy for emotion regulation, with this improvement partially mediating the facilitative effects of exercise on academic engagement and peer relationships (Rodriguez-Ayllon et al., 2019; Zhou et al., 2025). An exercise intervention study conducted by Pan et al. among Chinese children with learning difficulties found that a tailored exercise protocol not only improved executive function but also significantly enhanced emotion regulation capacity and social adaptation (Carcelén-Fraile et al., 2025; Xu Y. et al., 2026).

### 4.2 Response patterns of emotion-related brain regions to exercise

#### 4.2.1 Prefrontal-amygdala circuitry: the neural pathway underlying cognitive reappraisal

Top-down prefrontal regulation of the amygdala constitutes the core neural mechanism of emotion regulation, and exercise-induced enhancement of this circuitry represents one of the most critical neural pathways through which physical activity improves emotional health (Du et al., 2025; Tutakhail et al., 2026). fMRI studies have demonstrated that, during the performance of emotional face-matching tasks, chronically active adolescents exhibit significantly attenuated amygdala reactivity to negative emotional faces compared with their sedentary counterparts, accompanied by enhanced activation of the ventrolateral PFC (vlPFC) and dlPFC. Moreover, the strength of prefrontal-amygdala functional connectivity is significantly and negatively correlated with trait anxiety levels (Lakicevic et al., 2026; Tutakhail et al., 2026).

As with the neuroimaging findings in Section 3, these functional connectivity patterns are correlational (see Sections “2.2 Principal research paradigms and technical approaches” and “6.1.1 Interpretational limitations of resting-state functional connectivity”). The finding that exercise engagement is associated with attenuated amygdala reactivity and enhanced prefrontal-amygdala connectivity does not establish causation. Alternative explanations – including pre-existing differences in emotional processing, self-selection biases, or unmeasured confounders – cannot be ruled out. This remains a correlational observation requiring longitudinal and experimental validation.

Not all neuroimaging studies have reported significant exercise-related changes in prefrontal-amygdala circuitry. Several investigations have found null or non-significant associations between physical activity or fitness and amygdala reactivity or prefrontal-amygdala connectivity (Carcelén-Fraile et al., 2025; Lin et al., 2025). For instance, a study by Lakicevic et al. (2026) reported that while fitness was correlated with prefrontal activation during cognitive tasks, the association with amygdala reactivity during emotional face processing did not reach statistical significance (Carcelén-Fraile et al., 2025). Similarly, some cross-sectional studies have failed to find significant differences in amygdala volume or resting-state connectivity between active and sedentary adolescents (Richter et al., 2023). These null findings may reflect several factors: (a) small sample sizes limiting statistical power to detect moderate effects; (b) variability in emotion induction paradigms (e.g., face-matching vs. passive viewing vs. reappraisal tasks), which may differentially engage prefrontal-amygdala circuitry; (c) differences in exercise modalities and intervention durations across studies; and (d) individual differences in emotion regulation strategies that may moderate the exercise-affect relationship. The presence of null findings underscores that the association between exercise and prefrontal-amygdala connectivity, while promising, is not universally observed and requires further investigation with larger, more homogeneous samples and standardized protocols.

This finding has been interpreted as evidence that exercise may enhance the regulatory efficacy of prefrontal cortical regions over limbic emotional responses, thereby enabling individuals to more effectively “downregulate” exaggerated reactivity to negative emotional stimuli. The sensitivity of prefrontal-amygdala circuitry to exercise may be particularly pronounced during adolescence. During this developmental period, amygdala reactivity to emotional stimuli is at a relative peak, whereas prefrontal regulatory capacity remains incompletely mature. This developmental asynchrony – characterized by a “headstrong emotional engine and lagging cognitive brakes” – constitutes the neural substrate underlying heightened emotional volatility and increased risk-taking behavior in adolescence (Blakemore and Choudhury, 2006; Casey et al., 2008). Exercise may – in theory – accelerate the myelination and synaptic pruning of prefrontal-limbic connections, thereby narrowing this developmental mismatch window and engendering potentially protective neural adaptations. This hypothesis is supported by animal models showing BDNF-dependent myelination, but direct human evidence for BDNF-mediated myelination of prefrontal-limbic circuitry in pediatric populations is currently lacking (see Section “2.3.1 Interpretational note on peripheral BDNF as a surrogate marker” and Table 4 for detailed discussion of BDNF-related evidence limitations).

#### 4.2.2 Anterior cingulate cortex: a hub for emotional conflict monitoring and regulation

The anterior cingulate cortex (ACC) plays a pivotal role in emotional conflict monitoring and emotion regulation. The rostral ACC (rACC) is implicated in the evaluation of emotional salience and the detection of emotional conflict, whereas the dorsal ACC (dACC) is engaged in the recruitment of cognitive control resources. The effects of exercise interventions on these two subregions exhibit differentiated patterns. Following chronic exercise, the neural sensitivity of the rostral ACC to emotional conflict is enhanced – manifested as elevated activation during conflict trials in emotional Stroop tasks. Concurrently, functional connectivity between the dorsal ACC and dlPFC is strengthened, reflecting more efficient mobilization of cognitive control networks in the context of emotional conflict (Hoy et al., 2025; Tutakhail et al., 2026).

As a critical intersection node linking executive function and emotional networks, the exercise sensitivity of the ACC provides an integrative neural perspective for understanding why exercise simultaneously improves both cognition and emotion. Functional enhancement of this single neural structure may concurrently radiate to the domains of cognitive control and emotion regulation, thereby constituting the structural foundation for the cross-domain effects of exercise (Zelazo and Carlson, 2012).

#### 4.2.3 Insula and interoceptive processing: the neural substrate of embodied emotion regulation

Among the neural mechanisms discussed in this review, interoceptive processing – the neural representation of bodily states – provides the most direct empirical instantiation of embodied cognition mechanisms in the exercise context. As introduced in Section 2.1, embodied cognition theory posits that cognitive and emotional processes are grounded in sensorimotor experience. The insula, as the primary cortical hub for interoception, represents the neural interface through which bodily signals are transduced into conscious emotional experience and, potentially, into cognitive regulation. The findings reviewed below are consistent with – but do not directly test – the embodied cognition prediction that exercise enhances emotional regulation by improving the neural representation of bodily states. With this caveat in mind, we now turn to the empirical evidence on insula and interoceptive processing.

The insula constitutes the core neural substrate for interoceptive processing, responsible for receiving and integrating signals originating from within the body – including cardiac, respiratory, and visceral states – and relaying this information to the ACC and PFC for higher-order representation. The training effects of exercise on the interoceptive system are emerging as a novel focal point in research on embodied emotion regulation (Chaddock-Heyman et al., 2014b; Cotman et al., 2007; Gomez-Pinilla and Hillman, 2013; Ratey and Loehr, 2011).

Regular exercise training may enhance interoceptive sensitivity. Chronically active individuals exhibit greater precision in detecting their own heartbeat rhythms and respiratory states, and this improvement in interoceptive accuracy is significantly correlated with enhanced emotional awareness. Functional integration of the insula-anterior cingulate-prefrontal interoceptive network is strengthened following exercise interventions, thereby providing neural circuit-level support for the embodied mechanism of “regulating emotion through perceiving the body” (Li et al., 2025b; Shi et al., 2025). This perspective relocates the emotional benefits of exercise to the body itself – exercise does not improve mood indirectly through “endorphin release” or “distraction,” but rather directly alters the generative process of emotional experience by remodeling the neural representation of the interoceptive system (Pourteymour et al., 2025; Singh et al., 2025). Consistent with the caveats outlined in Sections “3 Physical exercise and executive function: effects and neural mechanisms” and “4.2.1 Prefrontal-amygdala circuitry: the neural pathway underlying cognitive reappraisal”, these interoceptive findings should be interpreted with caution. The observed associations between exercise, enhanced interoceptive accuracy, and improved emotional awareness are correlational and do not establish that exercise-induced insula changes cause emotion regulation benefits. Behavioral validation – demonstrating that improvements in interoceptive accuracy predict improvements in emotional functioning – is essential but has been conducted in only a limited number of studies. The neural findings reviewed here are therefore best understood as suggestive of embodied emotion regulation mechanisms rather than as definitive evidence of causation.

A recent study by Yao et al. employed virtual reality-enhanced aerobic exercise as an intervention for adolescents with depression and found that the emotional improvement effects of VR-enhanced exercise were significantly superior to those of traditional aerobic exercise. The underlying mechanism involved stronger activation of the insula-prefrontal interoceptive network. This finding introduces a novel, technology-augmented approach for optimizing the emotional benefits of exercise interventions – specifically, that amplifying bodily perceptual feedback during exercise (e.g., the visual-proprioceptive synchrony afforded by virtual reality) may potentiate interoceptive training effects (Peng et al., 2026; Yao et al., 2026). In summary, the empirical evidence on insula-mediated interoceptive processing is consistent with embodied cognition predictions, but direct tests of embodied cognition in pediatric exercise contexts are currently absent. The observed associations between exercise, enhanced interoceptive accuracy, and improved emotional regulation provide indirect support for the embodiment framework, yet they do not establish that bodily states are constitutive of emotional experience in the sense proposed by strong versions of embodied cognition theory. We therefore interpret these findings as suggestive of, rather than confirmatory for, embodied mechanisms, and we highlight the need for future studies specifically designed to test embodied cognition predictions in youth populations (e.g., comparing interoceptive training with traditional exercise protocols) (Figure 5).

**FIGURE 5:**
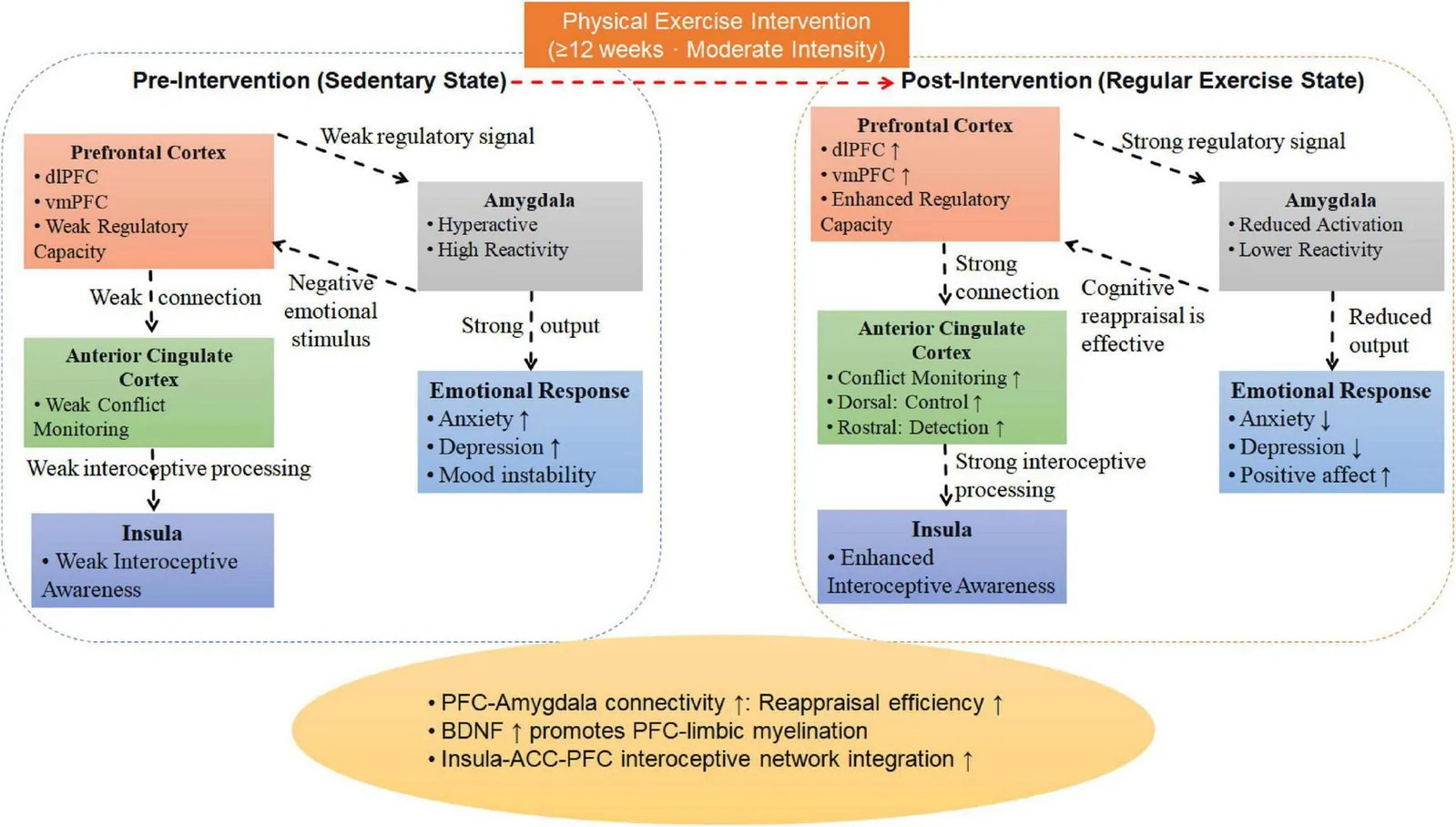
Physical exercise remodels the prefrontal-limbic emotion regulation circuit in youth. This Figure presents a comparative depiction of the prefrontal-limbic emotion regulation circuitry before (upper panel, sedentary state) and after (lower panel, regular exercise intervention lasting ≥ 12 weeks). Pre-intervention (sedentary state): The prefrontal cortex (dlPFC and vmPFC) exhibits weak regulatory capacity over subcortical emotion-generative regions. The amygdala shows hyperactivation and heightened reactivity to negative emotional stimuli. The anterior cingulate cortex (ACC) demonstrates weak conflict monitoring, and the insula exhibits poor interoceptive awareness of bodily signals (heartbeat and respiration). Consequently, emotional responses are characterized by elevated anxiety, increased depressive symptoms, and mood instability. Arrow annotations indicate weak top-down regulatory signals from prefrontal regions and strong bottom-up drive from negative emotional stimuli. Post-intervention (regular exercise state): Following ≥ 12 weeks of regular moderate-intensity exercise, the prefrontal cortex (dlPFC ↑, vmPFC ↑) shows enhanced regulatory capacity. Amygdala activation is attenuated and reactivity to negative stimuli is reduced. The ACC exhibits improved conflict monitoring, with the dorsal subdivision showing enhanced cognitive control and the rostral subdivision demonstrating heightened emotional conflict detection. The insula displays increased interoceptive sensitivity to cardiac and respiratory signals. Emotional regulation outcomes include reduced anxiety, decreased depressive symptoms, and elevated positive affect. Arrow annotations reflect strengthened top-down regulatory signaling and effective cognitive reappraisal. Bottom mechanism panel: Three key neurobiological mechanisms underlie this remodeling: (1) enhanced prefrontal-amygdala functional connectivity supporting more efficient cognitive reappraisal; (2) BDNF upregulation promoting myelination of prefrontal-limbic connections; and (3) optimized integration of the insula-ACC-prefrontal interoceptive network, which grounds emotion regulation in enhanced bodily awareness. dlPFC, dorsolateral prefrontal cortex; vmPFC, ventromedial prefrontal cortex; ACC, anterior cingulate cortex; BDNF, brain-derived neurotrophic factor.

### 4.3 Interaction between executive function and emotional brain regions: a cognitive-emotional coupling perspective

The preceding sections have separately addressed the effects of exercise on executive function and emotional brain regions. However, these two domains exhibit considerable overlap in both neuroanatomical organization and functional engagement, and are tightly intertwined in behavioral expression (Diamond, 2020; Zelazo and Carlson, 2012). The PFC serves as a “shared hub” for executive function and emotion regulation, with its functional status concurrently determining both the efficiency of cognitive control and the efficacy of emotional regulation. Enhanced activation of the dlPFC subserves not only working memory updating but also participates in the downregulation of emotion during cognitive reappraisal; the vmPFC is engaged in value-based decision-making while also modulating the intensity of amygdala-driven emotional responses (Bandettini et al., 2025; Singh et al., 2025).

This shared neural substrate constitutes the structural precondition for the “two birds, one stone” effect of exercise: functional remodeling of prefrontal-limbic circuitry induced by exercise simultaneously radiates to both cognitive and affective domains. More importantly, a bidirectional facilitative relationship exists between executive function and emotion regulation. Enhanced executive function furnishes more abundant cognitive resources for emotion regulation (e.g., more efficient working memory updating supports more flexible cognitive reappraisal), while improved emotional states establish optimal arousal conditions for cognitive performance (excessive anxiety impairs cognitive efficiency, whereas moderate positive affect facilitates cognitive flexibility). Exercise may thus generate synergistic effects that transcend single-domain interventions by concurrently activating these two mutually reinforcing adaptive pathways. A review by Chaddock-Heyman et al. systematically elucidated the relationship among cardiorespiratory fitness, cognitive control, and memory in children, noting that prefrontal-hippocampal circuitry constitutes the shared neural substrate through which exercise simultaneously influences cognition and emotion (Chaddock-Heyman et al., 2014b).

## 5 Moderating variables and boundary conditions

### 5.1 Exercise prescription parameters

Before summarizing the evidence on specific exercise prescription parameters, two important caveats warrant explicit acknowledgment. First, the parameters discussed below represent the modal characteristics of successful interventions identified across a heterogeneous literature – that is, the features most commonly observed among protocols that reported positive effects on cognitive or emotional outcomes. These should be interpreted as general guidance for intervention design rather than as precisely established optimal prescriptions, universally applicable optimal prescriptions. Second, the confidence with which each parameter can be recommended varies considerably. As summarized in Table 2, the evidence for moderate-intensity aerobic exercise and interventions lasting ≥ 12 weeks is relatively consistent (Moderate confidence), whereas the specific recommendation of exactly 45-min sessions rests on a thinner evidence base (Low confidence), with few studies directly comparing durations. Given the substantial variation across studies in age, baseline fitness, exercise modality, intervention fidelity, cognitive outcomes, and study quality, precise prescription parameters are not yet justified by the current evidence.

#### 5.1.1 Exercise type: differential effects

Exercise type constitutes a primary-order variable moderating the cognitive and emotional effects of physical activity. The neural circuits and molecular pathways activated by different exercise modalities exhibit both convergence and differentiation (Guzmán-Muñoz et al., 2025; Singh et al., 2026).

Aerobic exercise, by virtue of its robust effects on BDNF upregulation and cardiovascular adaptation, occupies a foundational position in the promotion of executive function (Dinoff et al., 2016; Huang et al., 2014; Szuhany et al., 2015). A review by Huang et al. confirmed that regular aerobic exercise significantly elevates BDNF levels, with effects further amplified following improvements in aerobic fitness (Szuhany et al., 2015). A meta-analysis by Dinoff et al. found that HIIT elicits superior BDNF upregulation compared with moderate-intensity continuous exercise, suggesting that intensity constitutes a critical dimension modulating the effects of exercise type (Dinoff et al., 2016).

Cognitively engaging physical activities have garnered particular attention in recent years. The core defining feature of these activities is the embedding of executive function recruitment within the movement task itself. A multilevel meta-analysis by Teng et al., encompassing 15 studies and 1,671 participants, found that cognitively engaging physical activities confer significantly superior effect sizes for executive function compared with traditional exercise protocols (Hedges’ g = 0.50; 95% CI not reported; a moderate effect). This effect size exceeds the overall effect of traditional exercise on executive function (g ≈ 0.35; small-to-moderate), suggesting that cognitive engagement during physical activity meaningfully enhances cognitive benefits (Teng et al., 2025). A meta-analysis by Mao et al. further corroborated this conclusion and revealed that cognitively engaging exercise exerts a larger effect size for inhibitory control (g = 0.58; a moderate-to-large effect) than for working memory (g = 0.41; a small-to-moderate effect) and cognitive flexibility (g = 0.37; a small-to-moderate effect) (Mao et al., 2024). A recent cluster randomized controlled trial validated the effectiveness of cognitively engaging physical activities within school settings, demonstrating significant improvements in executive function, mental health, and physical fitness among children in the intervention group (Xu . Y. et al., 2026). Research by Schmidt et al. found that classroom-based micro-breaks incorporating cognitive challenges yielded significantly superior facilitative effects on children’s attention compared with purely physical activity breaks (Schmidt et al., 2016).

Team sports and cooperative activities possess unique advantages with respect to emotional and social benefits. A systematic review by Eime et al. found that team sports confer significantly greater benefits for self-esteem, social skills, and sense of belonging compared with individual sports (Eime et al., 2013). Longitudinal studies by Vella et al. confirmed that participation in team sports predicts lower psychological difficulties and higher levels of social adaptation (Vella et al., 2015; Vella et al., 2017).

#### 5.1.2 Exercise intensity and duration: dose-response relationships

The relationship between exercise intensity and cognitive effects conforms to an inverted U-shaped profile. Moderate-intensity aerobic exercise is widely recognized as the “optimal window” for cognitive facilitation: intensities that are too low fail to trigger sufficient neurochemical responses, whereas excessively high intensities may offset or even reverse facilitative effects due to accumulated fatigue and depletion of cognitive resources (Du et al., 2025; González-Del-Castillo and Barbero-Alcocer, 2025; Li et al., 2025c; Xu et al., 2025). The classic investigation by Li et al. demonstrated that moderate intensity (60–69% of maximum heart rate) yields optimal facilitation of inhibitory control, low intensity (40–49%) is linked to only weak effects, and high intensity (80–89%) actually impairs performance (Li et al., 2025c).

The relationship between exercise intensity and cognitive outcomes is not unidirectional, and conflicting findings have been reported. While moderate-intensity exercise consistently shows facilitative effects, the evidence for high-intensity exercise is markedly inconsistent. Some studies have reported that HIIT produces superior BDNF upregulation compared with moderate-intensity continuous exercise (Drollette et al., 2014), whereas others have found that high-intensity exercise impairs or does not improve cognitive performance during or immediately following exercise (Mello et al., 2025; Willoughby and Hudson, 2023). For instance, Li et al. reported that switch costs in cognitive flexibility tasks increased following high-intensity exercise, suggesting a transient cognitive cost rather than benefit (Willoughby and Hudson, 2023). Tao et al. similarly found that high-intensity exercise impaired inhibitory control performance compared with moderate-intensity exercise (Mello et al., 2025).

Several explanations may account for these divergent findings. First, the timing of cognitive assessment is critical: high-intensity exercise may temporarily deplete cognitive resources during and immediately after exercise (the “catastrophe” or “overload” effect) but may confer benefits after a longer recovery period (Alvarez-Bueno et al., 2017; Mello et al., 2025). Second, baseline fitness level moderates the intensity-response relationship: individuals with higher baseline fitness may tolerate and benefit from higher intensities that are detrimental to less fit individuals. Third, the specific cognitive domain matters: high-intensity exercise may impair complex, resource-demanding tasks (e.g., cognitive flexibility, complex working memory) while leaving or even improving simpler tasks (Mello et al., 2025; Willoughby and Hudson, 2023). Fourth, dopaminergic and noradrenergic responses to high-intensity exercise may vary substantially across individuals due to genetic differences (e.g., COMT Val158Met polymorphism). Collectively, these factors suggest that the intensity-cognition relationship follows an inverted-U or inverted-J shape rather than a simple linear trend, and that high-intensity exercise may be beneficial for some outcomes, populations, and time points but detrimental for others.

With respect to duration, 45 min has been identified by multiple studies as the most frequently employed duration among protocols reporting positive effects. However, it is important to note that the evidence base for this specific duration is limited – few studies have directly compared different durations within the same population and outcome domain. The available evidence suggests that sessions of 20–45 min are commonly associated with positive outcomes, whereas sessions shorter than 20 min typically yield weaker effects and sessions exceeding 60 min may result in effect attenuation due to fatigue or motivational decline. Thus, 45 min should be understood as a modal value (i.e., the most common duration among successful protocols) rather than a precisely optimized duration derived from dose-response comparisons (Teng et al., 2025).

Regarding intervention duration, the preponderance of evidence suggests that interventions of at least 12 weeks are more consistently associated with positive cognitive outcomes compared with shorter interventions. It is important to note, however, that this threshold is derived from the modal duration among successful protocols, not from controlled dose-response comparisons, and studies with shorter durations (e.g., 6–8 weeks) have also reported positive effects on some outcomes. The 12-week threshold should therefore be understood as a general benchmark supported by the weight of evidence, but not as a strict minimum duration applicable across all populations, exercise modalities, and outcome measures. Longer interventions (e.g., ≥24 weeks) may yield more robust and enduring effects, although direct comparisons remain limited (Alvarez-Bueno et al., 2017; de Greeff et al., 2018; Singh et al., 2019).

Concerning frequency, the majority of successful intervention protocols have employed at least three sessions per week. While this frequency is commonly observed among positive-effect studies, direct evidence comparing two versus three versus five sessions per week is sparse, and the optimal frequency likely varies as a function of intensity, duration, and individual characteristics. The recommendation of “three sessions per week” should therefore be interpreted as a general guideline reflecting common practice in the literature rather than a precisely calibrated optimal frequency (Singh et al., 2019).

#### 5.1.3 Temporal dynamics of acute and chronic effects

Acute effects peak immediately post-exercise and may persist for 20–60 min before gradually returning to baseline (Chang et al., 2012; Ludyga et al., 2016). This temporal window suggests that scheduling moderate-to-vigorous physical activity prior to cognitively demanding learning tasks may yield short-term cognitive enhancement. The cumulative curve of chronic effects exhibits a progressive character: the initial 4–6 weeks are dominated by neurochemical adaptations (BDNF upregulation, altered neurotransmitter system sensitivity); functional connectivity reorganization begins to emerge between 6 and 12 weeks; and structural alterations become detectable only after 12 weeks or longer (Donnelly et al., 2016; Huang et al., 2014; Table 5).

**TABLE 5:** Summary comparison of acute versus chronic exercise effects on brain and cognition in youth.

Dimension	Acute exercise	Chronic exercise
Definition	Single bout of exercise, typically 20–60 min	Regular structured exercise over weeks to months (≥12 weeks typically required for structural changes)
Primary mechanisms	Neurochemical (catecholamine release, arousal ↑) + Hemodynamic (cerebral blood flow ↑, prefrontal oxygenation ↑)	Neurotrophic (BDNF ↑, IGF-1 ↑, VEGF ↑) + Structural (gray matter ↑, white matter integrity ↑) + Network reorganization
Neurochemical changes	Transient NE/DA release; BDNF surge (returns to baseline within 2–3 h)	Sustained BDNF elevation; basal BDNF ↑; TrkB signaling adaptation
Hemodynamic changes	Immediate CBF ↑; prefrontal oxygenation ↑ (20–60 min)	Capillary angiogenesis; basal perfusion ↑; cerebrovascular reserve ↑
Structural brain changes	None (within-session effects only)	GM volume ↑ (prefrontal, cingulate, hippocampus); cortical thickness ↑; WM integrity ↑ (DTI FA ↑)
Functional brain changes	P300 ↑, N200 ↓, ERN ↑ (immediate ERP changes); increased task-related activation	FPN connectivity ↑; DMN-FPN anti-correlation ↑; more focal/precise activation patterns
Cognitive effects	Immediate enhancement (peak 11–20 min post-exercise; effect size g = 0.2–0.5)	Stable improvements (effect size g = 0.35 for inhibitory control; cumulative over time)
Emotional effects	Immediate positive affect ↑; negative affect ↓ (effects persist 2–4 h)	Sustained emotional health benefits; depression/anxiety reduction (SMD = -0.48 to -0.62)
Temporal dynamics	Rapid onset; peaks within 20 min; returns to baseline within ∼60 min	Progressive: 0–6 weeks: neurochemical; 6–12 weeks: functional connectivity; ≥ 12 weeks: structural
Key methodological considerations	Timing of post-exercise assessment critical (peak effects brief); task selection important	Intervention duration, frequency, and fidelity critical; attrition common; long-term follow-up rare

BDNF, brain-derived neurotrophic factor; CBF, cerebral blood flow; DA, dopamine; DMN, default mode network; DTI, diffusion tensor imaging; ERN, error-related negativity; FA, fractional anisotropy; FPN, frontoparietal network; GM, gray matter; IGF-1, insulin-like growth factor-1; NE, norepinephrine; P300, P300 event-related potential; N200, N200 event-related potential; TrkB, tropomyosin receptor kinase B; VEGF, vascular endothelial growth factor; WM, white matter.

### 5.2 Individual difference variables

Exercise sensitivity differs between childhood and adolescence, a phenomenon closely associated with the gradient characteristics of regional brain maturation across developmental stages (Gogtay et al., 2004; Northey et al., 2025). The sensitive period hypothesis advanced by Naylor et al. posits that the development of specific executive functions may be characterized by temporal windows of heightened sensitivity to environmental stimuli, including physical exercise, during which exercise interventions may yield maximally facilitative effects (Northey et al., 2025).

Baseline functioning constitutes a critical variable for predicting the magnitude of exercise effects. Individuals with lower baseline executive function and higher baseline emotional symptoms typically exhibit greater intervention gains – a pattern characterized as “those who have the most to gain, benefit the most” (Cerrillo-Urbina et al., 2015; Den Heijer et al., 2017; Neudecker et al., 2019). This pattern carries positive implications for both clinical and educational practice: the populations most in need of intervention may, in fact, be those who derive the greatest benefit.

However, individual differences in exercise sensitivity must be interpreted in the broader context of potential confounding variables. Socioeconomic status, sleep, nutrition, pubertal status, and genetic background are among the factors that may either confound observed exercise-brain associations or moderate exercise effects in ways that are not yet well understood. These variables are discussed systematically in Section “6.1.4 Confounders and alternative explanations” and summarized in Table 6. The extent to which baseline functioning effects reflect genuine biological sensitivity versus these confounded influences remains an open question requiring further investigation.

**TABLE 6:** Methodological limitations of major meta-analyses cited in this review.

Meta-analysis	Focus	No. of studies (sample size)	Heterogeneity (I^2^)	Publication bias assessed?	Quality assessment used?	Key limitations	Implications for interpretation
Chang et al., 2012	Acute exercise → cognition	79 studies	50–70%	Funnel plot asymmetry observed	Not reported	Wide variation in exercise protocols; cognitive tasks; timing of assessment	Pooled effect (g = 0.2–0.5) may mask substantial variability; effects task- and time-dependent
Ludyga et al., 2016	Acute exercise → EF components	∼30 studies	Not fully reported	Limited	Not reported	Focus on moderate-intensity; limited high-intensity data	Facilitative effects of moderate-intensity most stable; high-intensity effects uncertain
de Greeff et al., 2018	Chronic exercise → EF/attention	∼30 studies	I^2^ ≈ 55–60% (WM); 40–45% (IC)	Not reported	Not reported	Broad age range; varied exercise protocols; limited WM studies	IC effects more consistent than WM; WM evidence weaker
Alvarez-Bueno et al., 2017	School-based PA → cognition	∼20 RCTs	Not fully reported	Not reported	Yes (PEDro)	School-based interventions vary widely; limited blinding	Curriculum-integrated PA shows promise; implementation fidelity unclear
Singh et al., 2019	PA interventions → cognition/academics	58 RCTs	Not fully reported	Not reported	Yes (expert panel)	Broad outcome measures; expert panel not systematic quality assessment	g = 0.35 overall; optimal: ≥ 12 weeks, ≥ 3 × /week; heterogeneity not fully quantified
Szuhany et al. (2015)	Exercise → BDNF	29 studies	I^2^ ≈ 45%	Funnel plot assessed	Not reported	Heterogeneous exercise protocols; BDNF assay methods vary (serum vs. plasma); mostly adults	Peripheral BDNF elevation is robust (g = 0.46); but peripheral-central correspondence remains uncertain
Dinoff et al., 2016	Exercise training → BDNF	∼30 studies	Notable heterogeneity	Not reported	Not reported	HIIT vs. MICT comparisons limited; assay variability	HIIT shows larger BDNF elevation (g = 0.52); but limited head-to-head comparisons
de Menezes-Junior et al., 2022	Exercise → BDNF in children/adolescents	∼15 studies	Moderate-to-high	Not reported	Yes	Limited pediatric studies; assay variability; no central BDNF measures	Peripheral BDNF elevation in youth; but 30–40% of individual studies report null findings
Vazou et al., 2019	CEPA → cognition	∼20 studies	I^2^ ≈ 50%	Not reported	Not reported	Few direct comparisons; definitional variability of “cognitive engagement”	CEPA shows superior effects (g = 0.42 vs. 0.28); but evidence is indirect
Teng et al., 2025	CEPA → EF	15 studies (N = 1,671)	Not fully reported	Not reported	Yes (multilevel)	Limited number of studies; publication bias possible	CEPA shows moderate effect (g = 0.50); but few head-to-head comparisons; publication bias concern
Carter et al., 2016	Exercise → depression (adolescents)	11 RCTs	I^2^ ≈ 55%	Funnel plot assessed	Yes	Small number of RCTs; depression measures vary	Moderate effect (SMD = -0.48); but limited RCTs and moderate heterogeneity
Bailey et al., 2018	Exercise → depression (adolescents/young adults)	17 RCTs	I^2^ ≈ 50%	Not reported	Yes	Includes young adults; depression measures vary	Moderate-to-large effect (SMD = -0.62); effects increase with duration

BDNF, brain-derived neurotrophic factor; CEPA, cognitively engaging physical activity; EF, executive function; HIIT, high-intensity interval training; IC, inhibitory control; MICT, moderate-intensity continuous training; PA, physical activity; PEDro, Physiotherapy Evidence Database scale; RCT, randomized controlled trial; SMD, standardized mean difference; WM, working memory. This table summarizes key methodological limitations of the major meta-analyses cited in this review. Heterogeneity (I^2^) values are reported where available from the original publications. Publication bias assessment and quality assessment methods are noted as reported or not reported in the original meta-analyses. Readers are encouraged to interpret pooled effect sizes in light of these limitations.

### 5.3 Population specificity: comparison between typically developing and neurodevelopmental disorder populations

Although the primary focus of this review is on typically developing populations, we include a brief discussion of ADHD and ASD because studies in these populations offer mechanistic insights that may inform understanding of exercise effects on executive function and emotional brain circuitry more broadly. However, findings from clinical samples should be interpreted with caution and may not generalize directly to typically developing populations.

The attention-deficit/hyperactivity disorder (ADHD) population represents a key clinical subgroup within cognitive neuroscience of exercise research. A meta-analysis by Cerrillo-Urbina et al. indicated that the effect sizes of exercise interventions on executive function in children with ADHD (g ≈ 0.5–0.8) exceed those observed in typically developing populations (Cerrillo-Urbina et al., 2015). Systematic reviews by Den Heijer et al. and Neudecker et al. have further corroborated this conclusion (Den Heijer et al., 2017; Neudecker et al., 2019). A systematic evaluation by Zhu et al. within the Chinese literature similarly identified significant exercise-induced improvements in executive function among children with ADHD (Liu R. et al., 2025). Pontifex et al. and Ludyga et al. have provided neurobehavioral evidence supporting these effects (Ludyga et al., 2017; Pontifex et al., 2013).

The autism spectrum disorder (ASD) population presents a distinct evidence profile. A meta-analysis by Sowa and Meulenbroek found that exercise interventions yield relatively pronounced improvements in emotional and social functioning among children with ASD (Sowa and Meulenbroek, 2012). A systematic review by Bremer et al. further confirmed that team sports and cooperative activities confer unique socioemotional benefits within the ASD population (Bremer et al., 2016). A longitudinal study by Tan et al. demonstrated that sports participation positively predicts neurocognitive development in children, with effects evident across different developmental stages (Tan et al., 2025). Lang et al. found that a history of sports participation is associated with superior speed of emotional facial recognition (Lang et al., 2025).

In summary, exercise benefits are modulated by interacting factors: optimal outcomes require cognitively engaging, moderate-intensity activity (∼45 min/session, ≥12 weeks), while developmental stage, baseline deficits, and neurodevelopmental status (ADHD vs. ASD) shape individual responsiveness. Notably, the findings for ADHD and ASD are derived from clinical samples and require cautious interpretation when generalized to typically developing populations. Collectively, these findings underscore the necessity of moving beyond generic exercise recommendations toward precision-based, individualized approaches that tailor exercise prescriptions to specific populations and desired outcomes (Figure 6).

**FIGURE 6:**
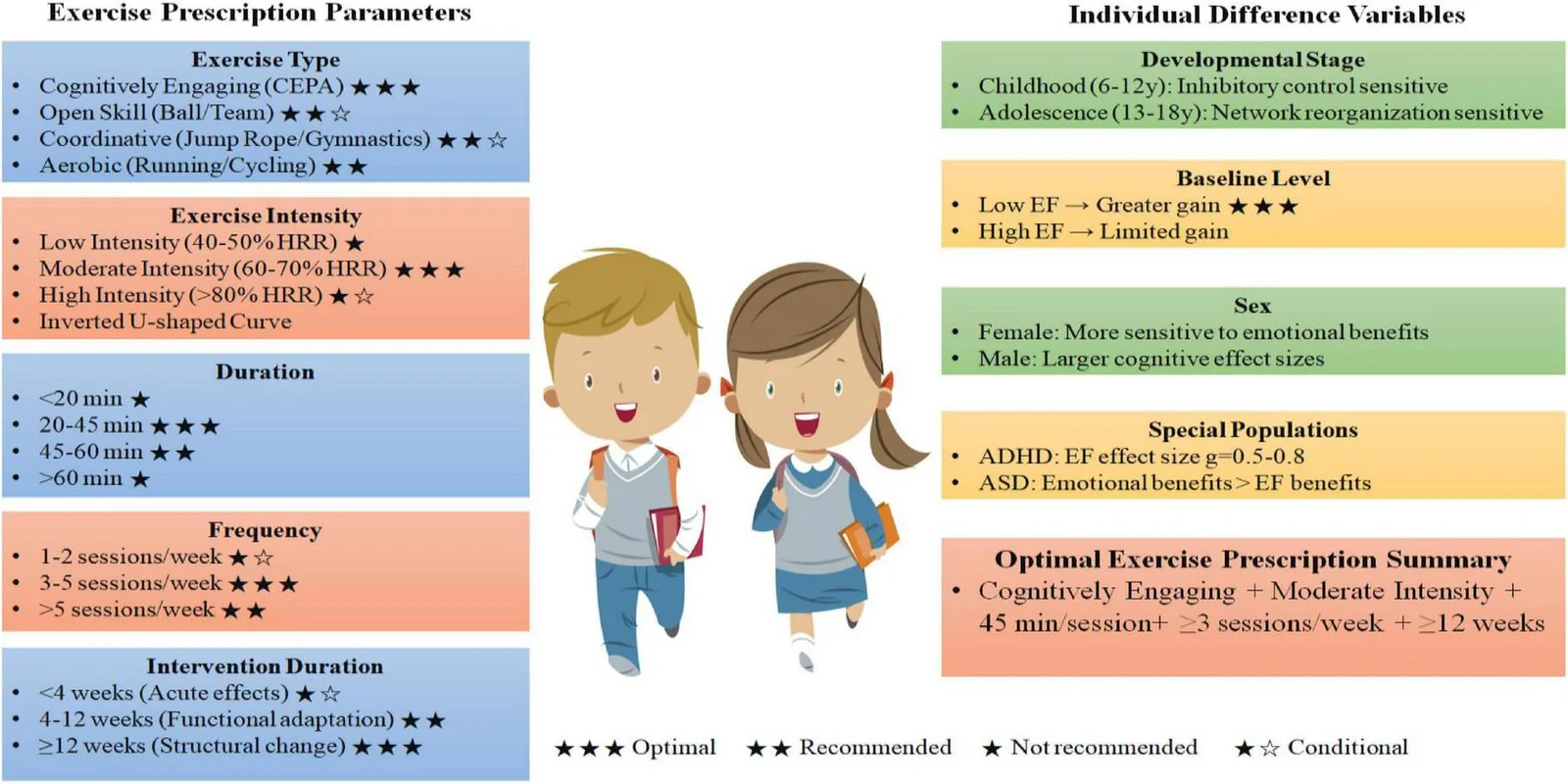
Moderators of exercise-induced cognitive and emotional benefits and optimal exercise prescription for youth. This Figure synthesizes evidence on key moderators of exercise effects and provides evidence-based recommendations for optimal exercise prescription. Upper panel: Exercise prescription parameters. The Figure summarizes the differential efficacy of exercise characteristics. Exercise type: Cognitively engaging physical activity (CEPA, ★★★) shows superior effects compared to open-skill activities (★★✩), coordinative exercise (★★✩), and aerobic exercise alone (★★). Exercise intensity: An inverted U-shaped relationship is observed, with moderate intensity (60–70% HRR, ★★★) producing optimal cognitive benefits, while low intensity (★) is insufficient and high intensity (★✩) may impair performance due to fatigue. Duration: Sessions of 20–45 min (★★★) are optimal; shorter (<20 min, ★) and longer (>60 min, ★) durations show reduced efficacy. Frequency: Three to five sessions per week (★★★) is recommended; one to two sessions (★✩) produce limited benefits, while more than five sessions (★★) may yield diminishing returns. Intervention duration: Structural brain changes require ≥ 12 weeks (★★★); 4–12 weeks (★★) is linked to functional adaptations, while < 4 weeks (★✩) captures only acute effects. Middle panel: Individual difference variables. Developmental stage: Childhood (6–12 years) shows heightened sensitivity for inhibitory control; adolescence (13–18 years) is sensitive to network-level reorganization. Baseline level: Individuals with lower baseline executive function show greater intervention gains (★★★), while those with higher baseline function have more limited improvement potential. Sex: Females may be more sensitive to emotional benefits, while males may show larger cognitive effect sizes. Special populations: Children with ADHD show larger effect sizes for executive function (g = 0.5–0.8) compared to typically developing peers; children with ASD show greater benefits for emotional regulation than for executive function. Bottom panel: Optimal exercise prescription summary. The current evidence supports a prescription of cognitively engaging physical activity at moderate intensity, delivered in 45-min sessions, at least three times per week, sustained for 12 weeks or longer, with individual characteristics modulating the magnitude of benefits. Prescription note: The optimal exercise prescription parameters summarized in this Figure represent the modal characteristics of successful interventions identified across a heterogeneous literature – the features most commonly observed among protocols reporting positive effects – rather than precisely established optimal prescriptions. Confidence in specific parameters varies: modality and intensity recommendations are supported by relatively consistent meta-analytic evidence (Moderate confidence), while the specific 45-min duration recommendation rests on a thinner evidence base (Low confidence), with few studies directly comparing durations. Individual characteristics and contextual factors should inform tailoring of these general guidelines to specific populations and settings. CEPA, cognitively engaging physical activity; HRR, heart rate reserve; ADHD, attention-deficit/hyperactivity disorder; ASD, autism spectrum disorder; EF, executive function; ★★★, optimal recommendation; ★★, recommended; ★, not recommended; ★✩ = conditionally applicable. Evidence note: The star ratings (★★★, ★★✩, etc.) in this Figure reflect the modal characteristics of successful interventions identified across the literature, not precisely established optimal parameters. Confidence levels for specific parameters are provided in Table 2 and range from High (inhibitory control outcomes) to Low (45-min duration recommendation). For detailed evidence grading of individual prescription parameters, see Table 2.

## 6 Research limitations and future directions

### 6.1 Limitations of current research

Despite the substantial progress documented in the preceding sections, several notable limitations constrain the interpretation and generalizability of the extant evidence base. First, considerable heterogeneity in exercise intervention protocols – encompassing variations in modality, intensity, duration, frequency, and delivery setting – renders cross-study comparison of effect sizes challenging and contributes to inconsistent findings across investigations. This methodological diversity, while reflecting the multifaceted nature of physical activity, impedes the derivation of standardized, evidence-based exercise prescription parameters. Second, self-selection bias constitutes a pervasive and serious confounding factor, wherein pre-existing individual differences in cognitive capacity, emotional regulation, or motivational disposition may drive both physical activity engagement and the outcomes of interest, thereby obscuring the true causal relationship. Third, as discussed in detail in Section “2.3.1 Interpretational note on peripheral BDNF as a surrogate marker,” the evidentiary link between peripheral BDNF and central neuroplasticity remains tenuous. Peripheral BDNF is an imperfect surrogate for central BDNF, and direct evidence for peripheral-central correspondence in pediatric populations is lacking. As noted in Section “2.3.1 Interpretational note on peripheral BDNF as a surrogate marker” and summarized in Table 4, the inferred pathway from peripheral BDNF to cognitive improvement remains a hypothesis requiring direct experimental validation. Fourth, the predominance of cross-sectional and short-term intervention designs limits causal inference regarding the enduring, developmentally cascading effects of exercise on brain structure and function across the transition from childhood to adolescence and into young adulthood. Fifth, the field has yet to fully embrace rigorous replication practices and open science frameworks, which constrains the accumulation of robust, reproducible knowledge. Finally, a significant portion of the neuroimaging evidence reviewed in this manuscript is based on small samples and has not been independently replicated. As detailed in Section “6.1.6 Interpretational caveats for neuroimaging biomarkers”, statistically significant neuroimaging changes should not be equated with functional cognitive improvement; behavioral correspondence is essential for meaningful interpretation. Many studies in this field report neuroimaging findings without adequate consideration of whether the observed neural changes translate into clinically or educationally meaningful behavioral benefits (Table 6).

#### 6.1.1 Interpretational limitations of resting-state functional connectivity

A substantial portion of the neuroimaging evidence reviewed in this manuscript derives from rs-FC (rs-FC) studies, which have provided valuable insights into the organizational properties of large-scale brain networks. However, several critical interpretational caveats warrant explicit acknowledgment to avoid overinterpretation of these findings.

*Biological heterogeneity of rs-FC changes.* Increased functional connectivity – whether within the FPN or between the FPN and DMN – does not uniquely index “improved neural efficiency” or “enhanced cognitive control.” Enhanced connectivity may reflect multiple distinct biological processes, including: (a) strengthening of synaptic efficacy and axonal myelination (genuine functional integration); (b) compensatory recruitment of additional neural resources (which may indicate inefficiency rather than efficiency); (c) reduced inhibitory signaling or decreased functional segregation; or (d) non-neural physiological confounds such as motion-related artifacts, respiratory variation, or cardiac pulsatility. Thus, the same observed increase in rs-FC is compatible with multiple, sometimes opposing, functional interpretations.

*Reproducibility and methodological sensitivity.* Graph-theoretical measures derived from rs-FC data, such as global efficiency, local efficiency, and modularity, have raised reproducibility concerns. Across independent datasets, the same graph-theoretical metric often yields inconsistent results due to differences in parcellation schemes, atlas selection (e.g., AAL vs. Power vs. Shen atlases), thresholding strategies, and the choice of correlation metric. The field has not yet converged on standardized analytic pipelines, and the generalizability of findings across preprocessing choices remains limited. The developmental context compounds this challenge, as the topological properties of brain networks undergo continuous reorganization throughout childhood and adolescence, and findings from adult populations cannot be directly extrapolated to developing samples.

*Preprocessing sensitivity.* rs-FC metrics are highly sensitive to preprocessing decisions. Motion correction is particularly consequential in pediatric samples, as children exhibit greater head motion than adults during scanning, and residual motion artifacts can produce spurious connectivity patterns that mimic legitimate neural signals. Global signal regression – a widely debated preprocessing step – removes variance shared across all voxels but introduces negative correlations and may artificially alter network anti-correlation patterns. The decision of whether to regress the global signal substantially affects findings regarding DMN-FPN anti-correlation, yet there is no consensus on the optimal approach, particularly in developmental populations where global signal may carry neural as well as physiological information.

Correlation is not mechanism. Perhaps most fundamentally, rs-FC findings are inherently correlational. The observation that higher fitness or exercise participation is associated with specific patterns of rs-FC does not establish that these connectivity patterns cause or explain improved cognitive performance. A comprehensive mechanistic account of exercise effects on the brain must integrate evidence across multiple levels – molecular, structural, functional, and behavioral – and cannot rely on rs-FC alone as evidence of “neural efficiency.” Without corresponding behavioral validation, neuroimaging biomarkers should not be interpreted as direct indicators of cognitive enhancement.

*Recommendation for interpretation.* Throughout this review, when citing rs-FC findings, we consistently interpret them as correlates of exercise engagement rather than as evidence of a causal mechanism. Where we describe associations between network metrics and cognitive outcomes, these are presented as observations requiring further mechanistic and experimental validation, not as established explanations of cognitive improvement.

#### 6.1.2 Publication bias and statistical heterogeneity

Several additional methodological limitations affect the interpretation and generalizability of the evidence reviewed above.

*Publication bias.* The published literature on exercise and cognition in youth is subject to publication bias – the tendency for studies reporting positive, statistically significant findings to be more likely published than those reporting null or negative results. Evidence for publication bias in this field comes from several sources: (a) funnel plot asymmetry observed in multiple meta-analyses, particularly for acute exercise studies (e.g., Chang et al., 2012; Ludyga et al., 2016); (b) the predominance of positive findings in the published literature despite known heterogeneity in individual responses to exercise; and (c) the relative scarcity of published null results or replication failures. This bias likely inflates the apparent consistency and magnitude of exercise effects. The reader should therefore interpret the positive conclusions of this review with the understanding that the published literature may not represent the full distribution of research findings, and that unpublished null results may temper the overall evidence base.

*Statistical heterogeneity.* The meta-analyses cited throughout this review consistently report moderate-to-high statistical heterogeneity (I^2^ values frequently ranging from 40% to 70%), indicating that the observed variation across studies exceeds what would be expected by chance alone. Sources of heterogeneity include: differences in exercise protocols (modality, intensity, duration, frequency), participant characteristics (age, baseline fitness, developmental status), outcome measures (different cognitive tasks purportedly measuring the same construct), study designs (RCTs vs. quasi-experimental vs. cross-sectional), and methodological quality. High heterogeneity reduces the certainty of pooled effect size estimates and suggests that the average effect may not apply uniformly across all populations and contexts. The confidence ratings provided in Table 5 account for this heterogeneity.

*Small sample sizes in neuroimaging studies.* A particularly notable limitation concerns neuroimaging studies, which typically employ small sample sizes (median n = 20–40 per group in many cross-sectional studies; n = 15–25 per group in RCTs with imaging components). These sample sizes provide limited statistical power to detect the modest effect sizes typically observed in exercise-cognition research (g ≈ 0.2–0.5). Small samples increase the risk of both: (a) false negatives (type II errors – failing to detect a true effect), and (b) false positives (type I errors – detecting an effect that is not replicable, particularly when multiple statistical comparisons are conducted without appropriate correction). The neuroimaging findings reviewed here should therefore be interpreted with caution and considered hypothesis-generating rather than confirmatory. Replication in larger, independently collected samples is essential before these findings can be considered robust.

*Patterns of divergent findings across the literature.* Beyond the statistical heterogeneity quantified by I^2^ values, several specific patterns of divergence merit explicit acknowledgment. First, working memory outcomes consistently show smaller and less consistent effect sizes than inhibitory control, with many individual studies reporting null effects even when inhibitory control improves (Donnelly et al., 2016; Hillman et al., 2014; Kamijo et al., 2011). This pattern may reflect the distributed neural substrate of working memory, which requires more extensive functional reorganization to manifest behavioral improvement. Second, high-intensity exercise effects are particularly inconsistent, with some studies reporting cognitive benefits and others reporting impairments (Drollette et al., 2014; Mello et al., 2025; Willoughby and Hudson, 2023). This divergence likely reflects the interplay of assessment timing, baseline fitness, and cognitive task demands. Third, BDNF findings are highly heterogeneous, with approximately 30–40% of individual studies reporting null effects despite a positive meta-analytic summary (Chang et al., 2012; Hillman et al., 2009; Liu Y. et al., 2025). This inconsistency may stem from methodological differences (serum vs. plasma assays, timing of blood sampling) and genetic moderation (BDNF Val66Met). Fourth, emotional neuroimaging findings are less consistently observed than behavioral emotional effects, with many studies failing to find significant prefrontal-amygdala connectivity changes even when self-reported mood improves (Carcelén-Fraile et al., 2025; Lin et al., 2025). This discrepancy highlights the indirect nature of neuroimaging measures and the possibility that emotional benefits of exercise are mediated by non-neural (e.g., psychosocial) pathways. Collectively, these patterns of divergence indicate that the exercise-brain-behavior relationship is domain-specific, protocol-dependent, and moderated by individual characteristics – and that the positive meta-analytic summaries, while important, should not be mistaken for uniform effects across all outcomes and populations (Xu B. et al., 2026).

*Implications for interpretation.* Given publication bias, high heterogeneity, and small sample sizes in many studies, the conclusions of this review should be understood as reflecting the best available evidence at the present time, with confidence varying substantially across specific conclusions (as summarized in Table 3). We explicitly avoid overgeneralizing from individual studies and instead base our conclusions on the convergence of findings across multiple independent investigations and meta-analyses, while acknowledging the limitations of the underlying evidence base.

#### 6.1.3 Methodological quality of included studies

A comprehensive assessment of methodological quality across all included studies is beyond the scope of this narrative review. However, several recurring methodological weaknesses warrant explicit acknowledgment. First, randomization and allocation concealment are not consistently reported or implemented, particularly in school-based intervention studies where cluster randomization is common but often inadequately described. Second, blinding of participants and intervention providers is inherently impossible in exercise interventions, and many studies do not employ blinded outcome assessors, introducing performance and detection bias. Third, attrition is a common problem in longer-term interventions, and few studies adequately handle missing data or conduct intention-to-treat analyses. Fourth, intervention fidelity is rarely systematically assessed – many studies report that an intervention “occurred” but do not document whether it was actually delivered as intended (e.g., whether participants achieved target heart rates, whether cognitive engagement components were implemented). Finally, measurement invariance across age groups and time points is rarely tested in developmental studies, raising questions about whether observed changes reflect true developmental or intervention effects versus measurement artifacts. These methodological limitations should temper confidence in individual findings and underscore the need for more rigorous study design and reporting in future investigations.

Finally, developmental measurement invariance – the assumption that cognitive tasks measure the same construct across different age groups – is rarely tested in the exercise-cognition literature. This is a particularly serious concern when comparing exercise effects across childhood and adolescence. A Flanker task administered to a 6-year-old, a 10-year-old, and a 15-year-old may tap different cognitive processes at each age: younger children may rely more heavily on general attentional resources, whereas older children may engage more specialized inhibitory control networks (Best and Miller, 2010; Dinoff et al., 2016; Tutakhail et al., 2026). Without evidence of measurement invariance, observed age-related differences in exercise effects could reflect changes in what the task measures rather than changes in exercise sensitivity. Future studies should incorporate measurement invariance testing and, where possible, use multiple tasks tapping the same construct to triangulate findings.

#### 6.1.4 Confounders and alternative explanations

A substantial portion of the evidence reviewed in this manuscript derives from observational and cross-sectional studies, which are vulnerable to confounding by variables that influence both physical activity/exercise engagement and the cognitive/emotional outcomes of interest. While randomized controlled trials provide stronger evidence for causal inference at the behavioral level, they are not immune to confounding, particularly when randomization is imperfect, attrition is differential, or blinding is compromised. Several key confounders and alternative explanations warrant explicit discussion.

*Socioeconomic status (SES) and the home environment.* SES is among the most pervasive confounders in exercise-brain research. Children from higher-SES families tend to have: (a) greater access to organized sports and safe spaces for physical activity; (b) better nutrition and healthcare; (c) more cognitively stimulating home environments; and (d) higher baseline cognitive test scores independent of physical activity. Failure to adequately control for SES may inflate observed exercise-cognition associations, as SES-related advantages in cognitive development may be misattributed to physical activity. While many studies adjust for SES using parental education or income as covariates, residual confounding remains a concern, particularly when SES is measured crudely or when within-SES variation is large.

*Sleep.* Sleep quality and duration are independently associated with both physical activity and executive function. Children who are more physically active tend to have better sleep (an association that may be bidirectional), and better sleep in turn supports prefrontal cortical function, attention, and memory consolidation. Without adequate control for sleep parameters, exercise effects on cognition may be partially or fully mediated by sleep – or, conversely, sleep-related cognitive benefits may be misattributed to exercise. Few studies in the exercise-cognition literature have systematically measured or controlled for sleep, representing a significant gap.

*Nutrition and dietary patterns.* Diet quality – particularly intake of omega-3 fatty acids, antioxidants, and micronutrients such as iron and zinc – directly influences brain development and cognitive function. Children with healthier dietary patterns tend to be more physically active (or vice versa), and both factors may independently contribute to cognitive outcomes. The interaction between diet and exercise on neuroplasticity (e.g., the role of omega-3s in facilitating BDNF signaling) is an emerging area of research, but few intervention studies have systematically controlled for dietary intake or examined diet-exercise interactions.

Obesity and body mass index (BMI). Overweight and obesity are associated with lower cognitive performance, reduced hippocampal volume, and altered prefrontal function. They are also associated with lower physical activity levels and reduced cardiorespiratory fitness. In cross-sectional analyses, associations between fitness and cognition may be partially or fully accounted for by BMI. While some studies adjust for BMI, few have examined whether BMI moderates or mediates exercise effects on cognition. Notably, some of the largest RCTs were conducted specifically in overweight children, which limits generalizability to normal-weight populations while also highlighting the importance of BMI as a moderator.

*Pubertal status.* Puberty is associated with profound neurobiological changes, including synaptic pruning, myelination, and alterations in dopaminergic and serotonergic systems – all of which affect executive function and emotion regulation. Pubertal status also influences physical activity patterns, with activity levels typically declining during and after puberty, particularly in girls. Cross-sectional studies that do not account for pubertal status (as opposed to chronological age) may confound developmental changes with exercise effects. The timing of puberty (early vs. late maturation) may also moderate exercise effects, an area that has received minimal empirical attention.

*Baseline intelligence and cognitive ability.* Children with higher baseline cognitive ability may be more likely to engage in organized sports (due to better motor learning, strategic understanding, or parental encouragement) and may also show faster cognitive development regardless of exercise. This introduces the possibility of reverse causality – higher cognitive ability leading to greater physical activity engagement rather than the reverse. Cross-sectional studies are particularly vulnerable to this confound, and even longitudinal studies with baseline adjustment may not fully eliminate reverse causality if unmeasured cognitive factors drive both activity and outcomes.

*School quality and educational environment.* School quality influences both opportunities for physical activity (availability of physical education, recess, sports teams) and cognitive outcomes (teaching quality, curriculum, academic expectations). School-level factors may thus confound associations between physical activity and academic/cognitive outcomes. Studies that do not account for school-level clustering or variability may overstate effects of activity on cognition by attributing school-related advantages to physical activity.

*Motivation and expectancy effects.* In exercise intervention studies, participants who volunteer for or are assigned to exercise interventions may have higher motivation, greater health awareness, or more positive expectations regarding the benefits of exercise – all of which could influence cognitive test performance independently of the physiological effects of exercise. Blinding of participants is inherently impossible in exercise trials, and while blinded outcome assessment can reduce detection bias, performance bias due to differential motivation or expectations cannot be fully eliminated. This is particularly relevant for studies using subjective or performance-based cognitive measures where motivation influences effort.

Cardiorespiratory fitness vs. physical activity: a recurring conceptual confound. As noted in Section 1.2, many studies use fitness as a proxy for activity, but fitness is influenced by genetics and maturation in addition to activity. Cross-sectional fitness-cognition associations cannot be interpreted as evidence for activity-cognition causation. Furthermore, randomized controlled trials of activity produce measurable fitness improvements but typically of smaller magnitude than the fitness differences observed in cross-sectional comparisons. Thus, the effect sizes observed in cross-sectional fitness studies may overestimate the benefits achievable through activity interventions.

*Genetic moderation.* The BDNF Val66Met polymorphism has been the most studied genetic moderator of exercise effects on cognition, with Met allele carriers sometimes showing greater BDNF responses to exercise (though findings are mixed). Beyond BDNF, dopamine-related genes – including COMT (catechol-O-methyltransferase) Val158Met and DRD2 (dopamine receptor D2) polymorphisms – may moderate exercise effects on prefrontal-dependent cognitive processes, given that exercise influences dopaminergic transmission. More broadly, exercise effects are likely moderated by polygenic profiles rather than single genes, but the evidence base for gene-environment interactions in pediatric exercise-cognition research remains extremely limited. Most studies are underpowered for genetic analyses, and replication is rare. Future studies with larger samples and genomic approaches are needed to systematically examine how genetic variation moderates exercise effects on brain and cognition.

*Summary.* The confounders and alternative explanations discussed above do not invalidate the conclusion that exercise benefits brain development, but they substantially qualify the strength and specificity of causal claims that can be derived from the existing evidence. Confidence in the exercise-brain association is highest for behavioral effects documented in well-controlled RCTs (e.g., exercise intervention → improved inhibitory control). Confidence is lower for mechanistic pathways inferred from correlational neuroimaging and biomarker studies, where the confounders listed above are more likely to be operating. Table 7 provides a summary of key confounders, their potential influence, and recommendations for future study design.

**TABLE 7:** Key confounders and alternative explanations in exercise-brain research.

Confounder	Direction of potential influence	Controlled in reviewed studies?	Recommended controls for future studies
Socioeconomic status (SES)	Higher SES → more physical activity + higher cognitive scores (inflates association)	Often adjusted (parental education/income), but residual confounding likely	Include multiple SES indicators; stratify or match by SES; use propensity score methods
Sleep	Better sleep → higher physical activity + better executive function (bidirectional)	Rarely measured or controlled	Objective sleep measurement (actigraphy); covary sleep parameters; examine sleep as mediator
Nutrition	Healthier diet → more activity + better brain development (likely confound)	Rarely measured	Dietary assessment (e.g., food frequency questionnaires); covary key nutrients (omega-3s)
Obesity/BMI	Higher BMI → lower activity + poorer cognition (confound; may also moderate)	Occasionally adjusted (BMI covariate)	Stratify by weight status; examine BMI as moderator; use BMI-matched controls
Pubertal status	Later puberty → more activity in some; puberty affects brain development (confound)	Rarely assessed; most studies use age only	Include Tanner stage or pubertal status measure; examine puberty × activity interactions
Baseline intelligence	Higher baseline IQ → more sports engagement + faster cognitive growth (reverse causality)	Sometimes adjusted (baseline performance)	Use longitudinal designs with multiple baseline assessments; apply change-score analyses
School quality	Higher-quality schools → more PE/sports + better academics (school-level confound)	Rarely accounted for	Multi-level modeling (students nested within schools); include school quality indices
Motivation/Expectancy	Higher motivation → greater intervention adherence + better test performance (performance bias)	Rarely measured	Assess and covary motivation/expectations; use active control conditions; minimize demand characteristics
Cardiorespiratory fitness (vs. activity)	Fitness reflects genetics/maturation as well as activity (conceptual confound)	Often conflated (fitness used as proxy for activity)	Distinguish fitness from activity conceptually; report both; interpret cross-sectional fitness findings cautiously
Genetic variation	BDNF, COMT, DRD2 polymorphisms moderate exercise effects (gene-environment interaction)	Rarely examined; studies underpowered	Larger samples for genetic analyses; polygenic approaches; replicate findings

#### 6.1.5 Practical significance of effect sizes

Throughout this review, we have reported effect sizes from meta-analyses and individual studies using standardized metrics including Hedges’ g, Cohen’s d, and standardized mean differences (SMD). To aid interpretation, it is useful to contextualize these effect sizes against conventional benchmarks and to consider their practical implications.

*Conventional magnitude benchmarks.* For Hedges’ g and Cohen’s d, conventional thresholds define small effects as g ≈ 0.2, moderate effects as g ≈ 0.5, and large effects as g ≈ 0.8 (Cohen, 1988). For correlation coefficients, small effects correspond to r ≈ 0.1, moderate to r ≈ 0.3, and large to r ≈ 0.5. For standardized mean differences (SMD) in meta-analyses of depression and anxiety, similar benchmarks apply, with SMD = 0.2–0.5 considered small-to-moderate and SMD = 0.5–0.8 considered moderate-to-large.

Interpretation of key findings. Exercise-cognition effect sizes predominantly fall in the small-to-moderate range: acute exercise (g = 0.2–0.5), inhibitory control (g = 0.35, 95% CI: 0.23–0.47), BDNF elevation (g = 0.46, 95% CI: 0.31–0.61), and depression reduction (SMD = -0.48 to -0.62). These are comparable to school-based interventions like class-size reduction (d ≈ 0.2) and peer tutoring (d ≈ 0.3–0.4).

*Practical significance.* In educational and clinical contexts, a small-to-moderate effect size (g = 0.2–0.4) corresponds to approximately a 2–4 percentile point improvement on standardized cognitive measures – modest at the individual level but potentially meaningful at the population level. For a classroom of 30 children, a g = 0.35 effect implies that the average child in the exercise group would outperform approximately 55–60% of children in the control group. Over a school year, such effects could translate into several months of developmental gain, particularly for children with lower baseline functioning. For depression and anxiety outcomes, a moderate effect (SMD = -0.5) corresponds to a clinically meaningful reduction in symptom severity, comparable to the effects of some psychotherapeutic interventions.

*Caveats in interpretation.* Several caveats temper the interpretation of these effect sizes. First, meta-analytic pooled estimates often mask substantial heterogeneity (I^2^ values ranging from 40% to 70% in many analyses), indicating that effects vary considerably across populations, protocols, and outcome measures. Second, publication bias may inflate pooled estimates, as null or negative findings are less likely to be published. Third, the effect sizes reported here reflect group-level averages and do not capture the substantial individual variability in response to exercise. Fourth, effect sizes for neuroimaging and biomarker outcomes are generally smaller and less robust than those for behavioral outcomes, reflecting the additional measurement noise and indirect inference involved in these modalities.

*Summary.* The exercise-cognition and exercise-emotion effect sizes reported in this review are consistently small-to-moderate in magnitude, comparable to other evidence-based educational and clinical interventions. While these effects are not “large” in the conventional statistical sense, their cumulative impact at the population level – combined with the low cost, low risk, and broad accessibility of exercise interventions – supports their practical significance for public health and educational policy.

#### 6.1.6 Interpretational caveats for neuroimaging biomarkers

A substantial portion of the evidence reviewed in this manuscript derives from neuroimaging studies – including task-based fMRI, resting-state fMRI, and event-related potentials (ERPs) – that have provided valuable insights into the neural correlates of exercise engagement. However, several critical interpretational caveats warrant explicit acknowledgment to avoid overinterpreting statistically significant neuroimaging findings as evidence of meaningful functional improvement.

Statistical significance does not equal functional significance. A statistically significant change in brain activation (e.g., increased P300 amplitude, enhanced prefrontal oxygenation, altered rs-FC) indicates that a measurable neural phenomenon has occurred with a probability greater than chance. It does not, however, demonstrate that this change translates into meaningful cognitive or behavioral improvement. Neuroimaging metrics are inherently indirect measures of neural activity, and their relationship to cognitive function is complex, non-linear, and context-dependent. Statistically significant neuroimaging findings should be interpreted as correlates of exercise engagement, not as direct evidence of cognitive enhancement.

The non-specificity of neuroimaging changes. Increased activation in prefrontal regions during a cognitive task – often interpreted as “enhanced neural efficiency” – can reflect multiple distinct biological processes: (a) genuine strengthening of task-relevant neural circuits; (b) compensatory recruitment of additional neural resources (which may indicate inefficiency rather than efficiency); (c) reduced inhibitory signaling or decreased functional segregation; (d) altered vascular reactivity rather than neural change; or (e) physiological artifacts such as motion or cardiac pulsatility. Without converging evidence from multiple modalities and experimental designs, a single neuroimaging finding cannot uniquely identify the underlying mechanism.

*The necessity of behavioral correspondence.* Perhaps most critically, neuroimaging findings should not be interpreted as evidence of cognitive enhancement in the absence of corresponding behavioral improvement. An increase in prefrontal activation during a working memory task, for example, does not establish that working memory has improved – only that neural activity has changed. If behavioral performance does not also improve, the functional significance of the neuroimaging change is ambiguous. Throughout this review, we have noted whether reported neuroimaging findings were accompanied by behavioral improvements; where such correspondence is absent, we have explicitly acknowledged this as a limitation.

*The risk of reverse inference.* Reverse inference – concluding that a particular cognitive process has occurred based solely on the pattern of brain activation – is a well-recognized pitfall in neuroimaging research (Poldrack, 2025). For example, observing prefrontal activation during a task does not uniquely identify “cognitive control” as the engaged process, as prefrontal regions are recruited by a wide range of cognitive operations. Similarly, observing exercise-related changes in prefrontal activation does not uniquely establish that “executive function has improved.” Such inferences require behavioral validation and are strongest when activation changes are accompanied by, and correlated with, behavioral improvements.

*Reproducibility and small sample sizes.* As noted in Section 6.1.2, neuroimaging studies typically employ small sample sizes (median n = 20–40), which are often underpowered to detect the modest effect sizes characteristic of exercise-cognition research. Small samples increase the risk of both false positives and inflated effect size estimates. Many neuroimaging findings in the exercise literature have not been independently replicated, and the field has been slow to adopt open science practices such as preregistration and data sharing. Consequently, the neuroimaging evidence reviewed here should be interpreted as hypothesis-generating rather than confirmatory.

*Summary.* Neuroimaging findings provide valuable, albeit indirect, evidence about the neural correlates of exercise engagement. However, statistically significant changes in brain activation or connectivity do not, by themselves, establish meaningful functional improvement. Behavioral validation – demonstrating that neuroimaging changes are accompanied by, and correlated with, improvements in cognitive performance – is essential for any functional interpretation. Throughout this review, we interpret neuroimaging findings with these caveats in mind, emphasizing that they are correlates of exercise engagement rather than direct evidence of cognitive enhancement (Table 4).

### 6.2 Future research directions

Future investigations should prioritize several strategic directions to advance the field beyond its current conceptual and methodological boundaries (Xu B. et al., 2026). First, concerted efforts should be directed toward the development of precision exercise prescriptions informed by individual neural profiles. This endeavor necessitates the identification of robust neuromarkers – derived from multimodal neuroimaging, electrophysiology, and genomics – that reliably predict differential responsiveness to specific exercise modalities and dosages, thereby moving the field from a “one-size-fits-all” approach toward tailored, mechanism-informed interventions. Second, the advancement and broader application of synchronized, multimodal neuroimaging acquisition protocols are imperative. Integrating techniques such as concurrent fNIRS and electroencephalography (EEG), or hybrid positron emission tomography-magnetic resonance imaging (PET-MRI) in pediatric populations where feasible, will enable more precise characterization of the spatiotemporal dynamics and neurochemical underpinnings of exercise-induced brain changes. Third, translational research that explicitly validates laboratory-derived evidence within authentic educational and community settings is urgently needed. The ecological validity of findings from controlled laboratory paradigms must be rigorously tested in the complex, real-world environments of schools and neighborhoods where youth actually live and learn. Fourth, the exploration of synergistic intervention protocols that strategically combine physical exercise with structured cognitive training warrants systematic investigation. Determining whether the concurrent or sequential pairing of these modalities yields additive or super-additive benefits for executive function and emotional regulation in both typically developing and clinical populations represents a promising frontier. Fifth, future research should be more deeply grounded within the theoretical framework of embodied cognition, advancing investigations into mind-body-brain integration. Critically, the field must move beyond interpreting findings as ‘consistent with’ embodied cognition and toward designing studies that directly test embodied cognition predictions. This includes: (a) testing whether interventions specifically targeting interoceptive awareness (e.g., yoga, mindfulness-based movement) produce larger emotion regulation effects than purely aerobic exercise; (b) examining whether individual differences in interoceptive sensitivity moderate the emotional benefits of exercise; and (c) using experimental manipulations that augment or attenuate bodily feedback during exercise to establish causal relationships between bodily signals and emotional outcomes. Such studies would help distinguish embodied cognition as a genuinely predictive scientific hypothesis from its current status in the exercise literature as a post hoc interpretive framework.

## 7 Conclusions and implications

### 7.1 Core conclusions

The preponderance of current evidence indicates that physical exercise is associated with beneficial effects on executive function and emotion regulation in youth. However, this conclusion is qualified by substantial heterogeneity in the evidence base. Not all studies report positive effects, and the strength of evidence varies considerably across cognitive domains (strongest for inhibitory control, weakest for working memory and cognitive flexibility), exercise intensities (moderate-intensity effects are most consistent; high-intensity findings are mixed), and outcome modalities (behavioral effects are more consistent than neuroimaging or biomarker effects). Null and contradictory findings are not uncommon, particularly in studies with small sample sizes, short intervention durations, or suboptimal assessment protocols. These inconsistencies should temper confidence in the universality of exercise benefits and underscore the need for continued investigation of moderating factors.

These effects are thought to be supported by multi-level neural mechanisms, although direct causal evidence for many proposed pathways remains incomplete (see Section “2.3 Macro-level pathway model of exercise effects on brain function” and Table 4). Among the three executive function components, meta-analytic evidence indicates that inhibitory control shows the largest effect sizes in response to exercise interventions, followed by working memory and cognitive flexibility. However, this ranking should be interpreted with several important caveats. First, as discussed in Sections 1.2 and 3, the three EF components are substantially intercorrelated in pediatric populations, and their neural substrates may be less differentiated in children than in adults. The apparent ‘sensitivity ranking’ may reflect task impurity, measurement invariance issues, or the frequency with which different EF components have been assessed, rather than selective neural targeting by exercise. Second, the ranking is descriptive at the group level and does not imply that individual children will show the same pattern of improvement. Readers are encouraged to interpret the differential sensitivity of EF components as a useful heuristic for understanding the existing literature, not as evidence for the neural separability of these components in developing populations.

Exercise may enhance the functional efficiency of prefrontal-anterior cingulate-parietal networks and strengthens top-down prefrontal regulation of amygdala reactivity, thereby concurrently optimizing cognitive control and emotional modulation. A core mechanistic triad – comprising BDNF upregulation, enhanced cerebral perfusion, and improved neural network efficiency – may underlie these behavioral and neurobiological benefits. It is important to emphasize that this triad is presented as a conceptual model (see Figure 2 and Table 4), not as a fully established biological sequence. For a detailed discussion of the limitations of peripheral BDNF as a surrogate marker, see Section “2.3.1 Interpretational note on peripheral BDNF as a surrogate marker.” It is important to emphasize that this triad is presented as a conceptual model (see Figure 2 and Table 4), not as a fully established biological sequence. The three pathways are not equally supported by evidence, and their proposed hierarchical relationship remains a theoretical proposition requiring direct experimental validation. When the existing literature is interpreted collectively, the modal characteristics of successful interventions – that is, the features most commonly observed among protocols reporting positive effects – include cognitively engaging, moderate-intensity physical activity, with session durations typically around 45 min, frequencies of at least three times per week, and intervention periods of 12 weeks or longer. However, as emphasized in Section “5.1 Exercise prescription parameters”, these parameters should be interpreted as general guidance for intervention design rather than as precisely established optimal prescriptions, and they require tailoring to individual characteristics and contextual factors. Several important qualifications apply: (a) substantial heterogeneity across studies in age, baseline fitness, exercise modality, intervention fidelity, cognitive outcomes, and study quality precludes highly specific, universally applicable prescriptions; (b) confidence in specific parameters varies considerably (see Table 2), with the 45-min duration recommendation resting on lower confidence than the modality or intensity recommendations; (c) these parameters should be interpreted as general guidance for intervention design and clinical translation, not as precise, fixed prescriptions; and (d) individual characteristics – including age, baseline cognitive status, and exercise preferences – should inform the tailoring of exercise protocols, moving beyond a one-size-fits-all approach toward precision-based, individualized interventions. For a detailed discussion of the practical significance of these effect sizes, including their interpretation as small-to-moderate effects and their comparability to other educational interventions, see Section “6.1.5 Practical significance of effect sizes”.

### 7.2 Implications for educational practice and policy

The accumulated neuroscientific evidence provides a foundation for public health and educational policy, although the certainty of this evidence varies across specific recommendations. While the general conclusion that physical activity supports brain development is well-supported, the evidence does not yet justify highly specific, universally mandated exercise prescriptions across all populations and contexts. The recommendations discussed in this review should therefore inform – but not rigidly constrain – the design of physical education curricula and public health guidelines, with continued adaptation based on emerging evidence and local implementation contexts. The recommendation that youth engage in no less than 60 min of moderate-to-vigorous physical activity daily is firmly grounded in a growing understanding of its salutary effects on brain development and mental health. The design and delivery of physical education curricula should be strategically reoriented to intentionally incorporate cognitively challenging elements – such as strategic decision-making, rule-switching, and cooperative problem-solving – to maximize the cognitive dividends of movement. Furthermore, the deepening and implementation of “integrated physical-educational” policies – which seek to embed physical activity across the school day and curriculum – require a more robust and directly applicable evidence base from the field of cognitive neuroscience of exercise to guide effective, scalable, and sustainable translation. Ultimately, positioning physical activity as an essential, non-negotiable component of the educational experience, rather than a discretionary or expendable add-on, represents a critical policy shift warranted by the converging evidence on exercise-dependent brain plasticity during this foundational developmental window.
